# Donor-specific, but not sex-specific, signatures dominate cell-type classification in lung scRNA-seq data

**DOI:** 10.1016/j.bbrep.2026.102692

**Published:** 2026-07-17

**Authors:** Nora Ghenciulescu, Marcel J. Reinders, Ahmed Mahfouz

**Affiliations:** a Pattern Recognition and Bioinformatics Group, TU Delft, Netherlands; b Department of Human Genetics, LUMC, Netherlands

**Keywords:** Single-cell RNA-seq, Classification, Sex effects, Donor effects, Lung

## Abstract

Transcriptomic differences between individuals and sexes are well-documented across tissues, affecting cell-type identity. Single-cell atlases often have skewed sex ratios or limited donor diversity, potentially leading to sex- or donor-biased annotations using automatic classification methods. This might cause models to exacerbate existing biases from their training data. We investigated this by varying the sex ratio in a training set and assessing a cell-type classifier’s performance on fixed single-sex test sets. To separate sex and donor effects, we ran the experiment with and without donor information. We found that differences between donors negatively impact classification, as evidenced by poorer performance on unseen donors. However, this is not primarily due to sex bias; the model classified male and female cells similarly well, even when trained on highly sex-skewed data. We also found that large sex-based abundance differences between cell types can confound performance interpretation, creating apparent sex-biased patterns. Our findings suggest that atlas creators and classifier developers must carefully consider donor-specific biases in scRNA-seq data.

## Highlights

• Donor variability, not sex, drives cell-type classification bias in lung data.
• Cell abundance differences can create false patterns of sex-specific performance.
• Cell type classifiers memorize donor-specific features, inflating accuracy on seen donors.
• Donor-based data splitting is vital to evaluate classifier fairness accurately.

## 1 Introduction

Inter-individual transcriptomic variability exists across human tissues and has been shown to manifest in cell type-specific ways [1], [2], [3]. An important dimension of this variation is the expression of sex-specific genes, driven by distinctions in gene regulation mechanisms between male and female cells, namely escape from X-chromosome inactivation for X-linked genes and hormone-related regulation of transcription factors for autosomal genes [4]. The extent of sex-based differential expression varies greatly between tissues [5], [6] and cell types [7], [8] and can affect tissue and organ morphology [9], [10], immunity [11], drug responses [12] and pathophysiology [13], [14], [15], [16]. Although the lung does not exhibit a particularly sex-specific transcriptomic signature, studies have linked variations in lung disease susceptibility and development to sex-specific transcriptomic differences, such as in the case of chronic obstructive pulmonary disease [17], [18], COVID-19 [14], inflammatory lung diseases caused by air pollution [19], and lung cancer [20], [21], [22], [23].

Bulk RNA sequencing (RNA-seq) methods have previously been used to investigate individual and sex-specific gene expression patterns at the tissue level throughout the human body [4], [5], [6], [24], [25]. However, bulk analyses are known to obscure inter-individual and sex-based variability [26], [27], [28], [29], particularly when there are differences in cell type abundance. As a result, single-cell RNA sequencing (scRNA-seq) methods are increasingly being used to profile gene expression at the cellular level, enabling the in-depth characterization of the cellular composition of human tissues [30]; such datasets can subsequently be used to investigate sex-dimorphic patterns of gene expression [31], [32].

Cell type annotation is a critical step in most scRNA-seq analysis pipelines, not only for individual datasets, but also to harmonize cell labels for dataset integration or to map new datasets to a reference atlas. A growing number of classification methods have been developed to predict cell labels, using pre-annotated datasets or reference atlases for training [33], [34], [35], [36]. These annotation methods have been compared on the basis of their sensitivity to the number of cells and cell types, population imbalance, input features, sequencing depth, scalability and computational efficiency [33], [34], [37], [38], yet there has been little focus placed on how they handle inter-individual variance or generalize under conditions of sex imbalance.

It has been hypothesized that machine learning models used in the single-cell analysis pipeline, including in the annotation step, could perpetuate biases in their training data that stem from unmeasured covariates or unbalanced population compositions [39]. This concern is particularly relevant in the construction of cell atlases, where some cell populations may originate from only a few donors or be dominated by a single sex, if the constituent datasets do not exhibit large enough donor diversity [40]. As a result, we propose that cell identification could become confounded with inter-individual variability or sex differences, yielding less accurate annotation of cells of unseen donors or of the minority sex. Consistent with this concern, donor origin has been shown to cause bias in the deconvolution of bulk RNA data using pseudobulk data obtained from scRNA-seq datasets as ground truth [41]. Automated annotation methods have also been shown to exhibit a drop in performance when tested on datasets that were not seen during training [33], but it is difficult to disentangle to what extent this is driven by donor variability as opposed to technical differences between datasets. At the bulk level, a transcriptomic aging ‘clock’ based on bulk expression data exhibits performance loss when trained on a single-sex dataset and predicting age in the opposite sex [42]. Similar examples of sex bias amplification have been described for other larger-level classifiers in the medical field [43], [44], [45].

Here, we investigate whether sex or donor biases in scRNA-seq data influence cell type classification. We focus on one of the largest tissue atlases to date, the Human Lung Cell Atlas (HLCA) [46], to identify cell types and subtypes that exhibit sex- or donor-based differences. First, we show that sex bias has minimal impact on cell-type classification in lung scRNA-seq data, with performance being largely comparable between male and female cells. Second, we find donor-specific transcriptomic signatures to be a major source of variation, reducing classifier accuracy on unseen individuals. Third, we show that differences in cell-type abundance can confound the interpretation of classifier performance, giving the appearance of sex-specific effects. Overall, these results highlight that, while cell type classifiers can robustly distinguish most lung cell types, careful consideration of donor composition and cell abundance is important for accurate evaluation and integration of single-cell datasets.

## 2 Results

### 2.1 Cell sub-populations in the HLCA show significant sex imbalance in abundance

**Fig. 1:**
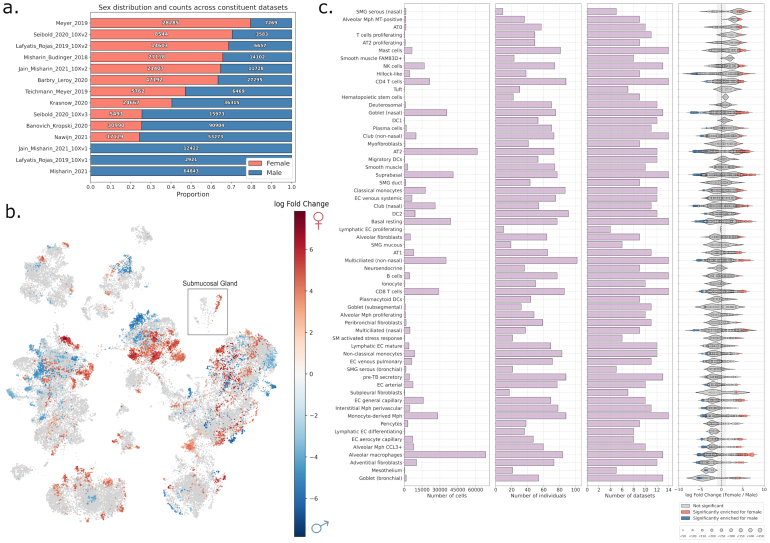
**a.** Sex distribution of the constituent datasets of the HLCA core, with cell counts given inside the bars. **b.** Abstracted graph of neighborhoods as found by Milo, superimposed onto the UMAP embedding of the HLCA core. The layout of nodes is determined by the position of the index cell in the UMAP. The neighborhoods displaying significant differential abundance, under an FDR threshold of 0.05, are colored by their log-Fold Change; red indicates significantly enriched in female samples and blue in male samples. Non-significant neighborhoods are shown in gray. Submucosal gland (SMG) cell population is highlighted. **c.** For each cell type at the finest annotation level, from left to right: the number of cells, the number of donors, the number of datasets and the distribution of differentially abundant neighborhoods as resulted from the differential abundance analysis. For the latter, each dot represents a neighborhood, colored in red if it is enriched in female samples and blue if it is enriched in male samples; neighborhoods colored in gray do not pass the significance threshold FDR <0.05. The size of the dots corresponds to the size of the neighborhoods, according to the legend. Cell types are ordered based on their median log Fold Change. The area shaded in gray indicates the region where the absolute log Fold Change is below 1 - cell types whose median log Fold Change falls within this region are considered sex-balanced, Supplementary Table 1.

The HLCA core consists of scRNA-seq samples collected from 107 individuals, across 14 datasets. While the overall male–female distribution is relatively well-balanced (60.5% male to 39.5% female), individual cell populations exhibit skewed sex ratios, particularly at finer annotation levels. This is due to the fact that several constituent datasets of the HLCA core are single-sex or unbalanced in terms of male–female distribution (Fig. 1(a)). To investigate the sex-based composition of the HLCA core, we perform differential abundance analysis using Milo [47], in order to identify cell neighborhoods with significant differences in male and female sample abundance. We find neighborhoods of cells that exhibit differential sex abundance across almost all cell types in the HLCA core (Fig. 1(b)), with notable variation in both neighborhood size and level of significance (Fig. S1(a),(b)). Neighborhoods strongly enriched for one sex tend to cluster together — for instance, within the submucosal gland (SMG) cell population, a distinct lobular region is composed almost entirely of female-dominated neighborhoods.

In order to analyze the differential sex abundance of individual cell populations, we assign cell type labels to each neighborhood according to the majority label among its constituent cells (Fig. 1(c)). This approach is valid since most neighborhoods are comprised of cells that belong to the same HLCA annotation (Fig. S1(c)). As a measure of sex-based abundance imbalance, we compute the female-to-male median log-fold change (Supplementary Table 1). We consider cell types with a negative median log-fold change to be male-dominated and cell types with a positive median log-fold change to be female-dominated. Cell types with an absolute median log-fold change below 1 are considered sex-balanced. We find that several cell types exhibit pronounced differences in abundance based on sex, with distributions skewed completely towards male or female (e.g., nasal serous SMG cells, MT-positive alveolar macrophages, bronchial goblet cells, mesothelial cells). Other cell types include both male- and female-dominated neighborhoods, yet their overall distribution is not skewed one way or another (e.g., basal resting cells, suprabasal cells, non-nasal multiciliated cells). These patterns generally align with the overall sex distribution across cell types (Fig. S2(a)), though differences are expected since neighborhood-level analysis captures local abundance patterns, rather than bulk proportions of cells.

For certain cell types, strong abundance trends may be partially caused by biases in the sampling procedure, such as large differences in the number of cells between cell types (Fig. 1(c)) or a lack of diversity in the contributing donors and datasets (Fig. 1(c), Fig. S2(b)). For example, the five populations that exhibit the largest skew towards female (nasal serous SMGs, MT-positive alveolar macrophages, AT0 cells, proliferating T cells and proliferating AT2 cells) all have lower cell counts than average, whereas high cell counts and originating from a large number of individuals and datasets generally yield balanced distributions (e.g., AT2 cells, basal resting cells, suprabasal cells). Some populations do not exhibit a skew in spite of their low cell counts, as long as there is a large enough diversity of datasets and donors (e.g., DC2 cells, B-cells, non-nasal club cells). However, these are not the only factors contributing to differential sex abundance — for instance, alveolar macrophages have the largest cell counts and originate from a large number of datasets and individuals, yet are still skewed towards male in distribution.

**Fig. 2:**
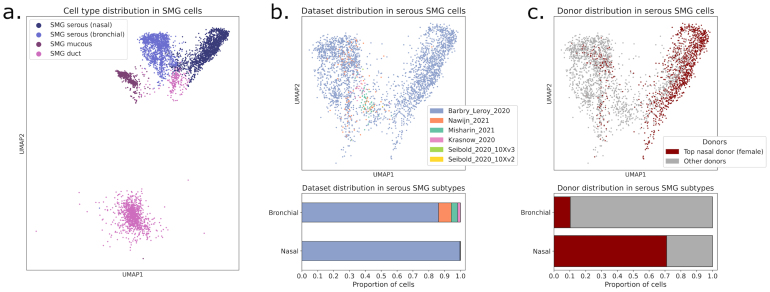
Exploration plots for the SMG cell population. **a.** Distribution of cell sub-types, as UMAP. The bi-lobular cluster in b. is mostly made up of serous SMG cells (nasal and bronchial). **b.** Distribution of datasets in the serous SMG sub-type, shown as UMAP and barplot. The ‘Barbry-Leroy’ dataset dominates. **c.** Distribution of donors in the serous SMG sub-type, shown as UMAP and barplot. The top nasal donor accounts for 70% of nasal samples (and 10% of bronchial samples).

One example of an unevenly sex-distributed dataset and donor distribution contributing to differential abundance is that of the SMG cell population (Fig. 2). The bi-lobular structure identified in Fig. 1(b), where one cluster is female-dominated, almost entirely consists of serous SMGs (Fig. 2(a)). Moreover, the female-associated cluster is mostly made up of nasal serous SMGs. Upon closer inspection of the serous SMG sub-population, we find clear disproportions in the contributing datasets and donors of origin (Fig. 2(b),(c)): one dataset (‘Barbry-Leroy 2020’) dominates, especially among the nasal sub-type, and a single (female) individual accounts for over 70% of the nasal cells which make up the female-skewed lobe. It is therefore difficult to determine whether the distinct distribution of nasal and bronchial cells is a consequence of cell type-specific differences in gene expression, or rather driven by sex- or donor-specific transcriptomic signatures.

The original integration in the HLCA was performed using scANVI [48], which simultaneously corrects for batch effects and computes a latent embedding of the data. The batch correction was performed at the dataset level, rather than for individual donors. As such, donor-specific transcriptomic variation may persist in the generated embedding. Given that some datasets are dominated by few donors or a single sex, the concern arises that such residual donor effects could manifest as apparent sex-specific clustering, for instance as described for the SMG population above. More broadly, since cell type annotations are derived from this corrected embedding, any such entanglement of donor and sex effects at the integration stage could confound cell type identity.

To investigate this, we performed independent batch corrections within the SMG population using Harmony [49], separately correcting for dataset effects and donor effects. Under the dataset-level correction, sex-based clustering is apparent (Fig. S3(a)–(c)), mirroring the original HLCA integration. However, when correcting for donor identity, this clustering is substantially reduced (Fig. S3(d)–(f)). These results suggest that the sex-specific patterns observed in the HLCA (Fig. 1(b)) are at least partly driven by inter-individual differences that are not resolved by the original HLCA integration, rather than reflecting sex-specific biology.

Given that differences in abundance exist not only in the SMG population, but across most cell types in the HLCA, the question arises whether sex or donor effects are confounding cell type identity during a classifier’s learning process and therefore bias data annotation. Since sex and donor effects are difficult to separate at the level of the cell type labels themselves, we consider both sources jointly when investigating classifier behavior.

### 2.2 Cell type classification in the lung is influenced by donor variation, not sex-specific effects

**Fig. 3:**
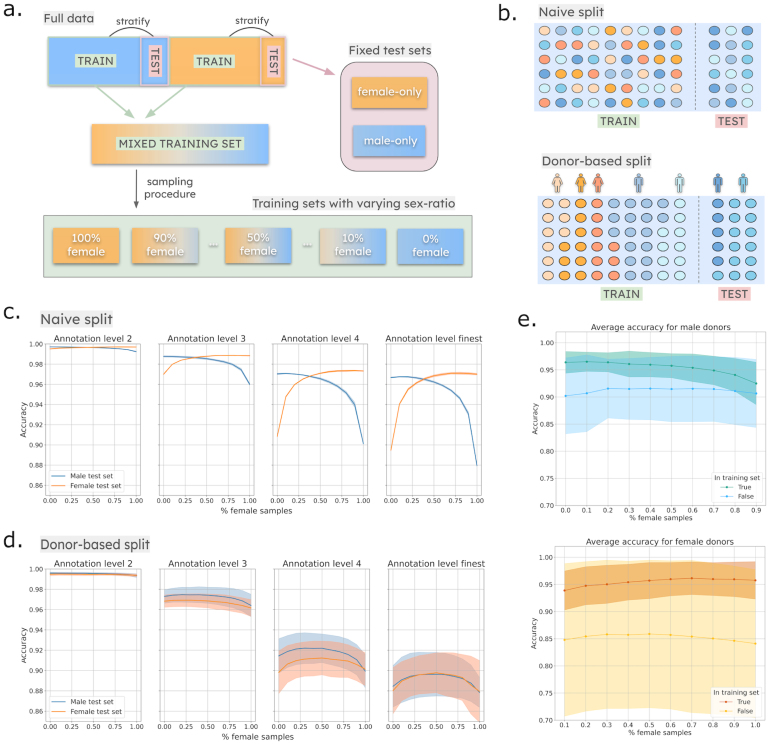
**a.** Schematic of the classification experiment. The full dataset is split into a male and female subset, then test sets are extracted for each sex, while stratifying for cell type with the respective training set. The two training sets are then combined into one. Using a semi-random sampling procedure that selects cells in adherence to an input sex ratio, we create 11 training sets that vary in male-to-female ratio from 0–100 to 100–0. The performance of the model, trained on each set, is subsequently assessed on the single-sex test sets extracted at the beginning of the procedure, which remain fixed throughout the experiment. **b.** Schematic of the filtering process, shown in the case of a 50–50 male–female split in the training set and evaluation on the all-male test set. Color coding indicates cells that come from a specific individual. In the naive split setting (above), there is no donor filtering: all cells are randomized into train and test set, regardless of donor origin; as a result, there are (male) donors that contribute to both the training and the test set. In the donor-based split (below), donor filtering is performed: individuals included in the test set cannot participate to the training set. **c.** Average accuracy of the KNN classifier on the male (blue) and female (orange) test set, in the naive split setting. X-axis shows the proportion of female cells in the training set, y-axis shows the accuracy score. The full line represents the mean accuracy across random seeds, the shaded area is the variance. **d.** As in c., for the donor-based split. **e.** Verification experiment showing that donor overlap introduces bias into the classification procedure. Train–test splitting and evaluation is performed as per the naive split setting for two thirds of the donors, while the other third are withheld and performance is evaluated on them separately. Average accuracy of the KNN classifier for male donors (above) and female donors (below). Color-coding indicates whether the donors contribute to the training set (shown in green for the male set and orange for the female set), or were excluded from training (shown in blue and yellow, respectively). Axes, full line and shaded area as in c.

To investigate how sex bias in the training set affects a cell type classifier’s performance, we train a K-Nearest Neighbors Classifier (KNN) to annotate cells into cell types based on the embedded gene expression data, under the following set-up: the sex imbalance ratio in the training set is gradually varied from 0% to 100% female, while evaluation is performed on fixed, single-sex test sets (Methods, Fig. 3(a)). Since annotation in the HLCA is performed hierarchically and we have access to labels across all levels, we perform training and testing across annotation levels 2-5. We establish two experimental settings (Fig. 3(b)), in terms of train-test splitting. In the naive split scenario, cells are randomized into a training set and a test set regardless of donor origin, according to the scheme in Fig. 3(a). As a result, the donors overlap between training and test set across all iterations of the experiment (Supplementary Table 2). We also employ a donor-based split, where donors are filtered such that no test data comes from individuals seen by the model during training — this is done in order to disentangle donor- and sex-specific effects as confounding factors for classification.

For the naive split scenario, the results of the classification experiment across annotation levels are shown in Fig. 3(c). It appears that the performance on the female-only test set increases as the proportion of female samples in the training set increases, while the performance on the male-only test decreases (and vice versa). This gap in accuracy is larger at finer annotation levels (8.7% drop on the male test set and 7.5% on the female test set), likely because of the increased difficulty of the classification task [33] and the finer-level cell populations being more differentially abundant across sexes. These results seem to indicate a sex bias: a classifier trained on female-dominated data does not perform well on male cells and vice versa. However, repeating the experiment on the donor-based data split, the sex bias effect disappears (Fig. 3(d)), with model performance following the same trend for the male and female test set, across sex proportions and annotation levels. Moreover, the overall accuracy drops in the donor-based setting as compared to the naive setting - for instance, at the finest annotation level, mean accuracy decreases from approximately 97% for the naive split (on both test sets, when the training set is sex-balanced) to below 90% in the donor-based split. To ensure robustness to class imbalances and different data representations, all results are verified by additionally calculating the F1 scores for the KNN classifier, as well as using a Random Forest classifier on the non-embedded data (Figs. S4–S7).

The absence of apparent sex-biased behavior in the donor-based split setting suggests that, due to donor overlap between the training and test set in the naive setting, the differences in performance on the male and female test set are, in fact, driven by individual-specific and not sex-specific differences in expression. When trained on cells from a given individual, the model likely memorizes donor-specific transcriptomic features and will therefore perform better on test cells from the same individual; if results are aggregated over sex, under skewed sex proportion, this effect can falsely appear sex-based, as in Fig. 3(c). To further confirm that naively splitting the data introduces bias into the experiment, we perform the classification again as in the naive setting, but withholding one third of the donors for testing (Fig. 3(e)). The performance is overall higher on donors included in the training set, indicating that the classifier indeed memorizes individual-specific features and donor contribution to the training therefore inflates model accuracy.

Given these results, donor-specific transcriptomic signatures appear to affect cell type classification, with the model performing worse on cells from previously unseen donors. Moreover, this drop in performance is not explained by sex-specific effects: when data splitting is performed in a donor-aware manner, average classifier performance is not affected by skewed sex distributions in its training set, even at finer annotation levels where we previously found differentially abundant patterns between the sexes.

### 2.3 Sex-specific classification differences at the individual cell-type level disappear when controlling for donor overlap and other confounders

To investigate classification patterns under sex and donor effects in more detail, we look at the classification performance of individual cell types at the finest annotation level, in relation to their abundance in the training set. This analysis is exploratory and aimed at describing observed patterns, not providing formal inferential evidence. We categorize cell types as exhibiting a ‘distinct’ or a ‘non-distinct’ trend across the two sexes, based on changes in the classification performance with respect to the proportion of female and male cells in the training data (see Methods for details). A cell type is distinct if prediction performance for that cell type shows a clear dependence on the sex skew of the training data and non-distinct if this is not the case; for cell types denoted as inconclusive, this could not be assessed due to low test counts or erratic classifier behavior.

In the naive split setting, cell types are evenly divided between the categories (Supplementary Table 3): 24 cell types exhibit distinct behavior, replicating the overall classification results (Fig. 3(c)), 20 cell types exhibit non-distinct behavior and 17 cell types are marked as inconclusive. As for the overall results (Fig. 3(c),(d)), this apparent sex-specific effect largely disappears when we move to the donor-based split setting, where only two cell types categorized as ‘distinct’ in the naive split setting maintain this categorization: AT0 and bronchial goblet cells (Supplementary Table 4, Fig. S8(a)–(d)). For all other cell types, sex-distinct classification trends are resolved under donor-level stratification (examples in Fig. S9).

The persistence of a sex-distinct classification trend for AT0 and bronchial goblet cells under the donor-based setting raises the question of whether the classifier is leveraging meaningful biological signal or is instead driven by sex-correlated demographic confounders. If male and female donors differ systemically in covariates such as age, tissue processing site or smoking history, failing to control for these could produce a spurious association between sex and classifier performance. To assess this, we repeated the classification experiment for AT0 cells and bronchial goblet cells, matching male and female donors on smoking status and tissue processing site; matching on age was not feasible due to limited availability of this covariate and its skewed distribution in the HLCA core. After matching, the classification trend on AT0 cells is no longer distinct (Fig. S8(e), Supplementary Table 5), suggesting that the observed effect was indeed driven by confounding. For bronchial goblet cells, cell counts in both training and test set were too low after matching to reliably assess classifier performance (Fig. S8(f), Supplementary Table 5).

Overall, these results suggest that, as for the overall experiment (Fig. 3(c),(d)), the effect of donor-specific transcriptomic differences on cell type classification is largely not explained by sex-based differences, given that most cell types behave non-distinctly after donor-level stratification and confounder matching.

### 2.4 Abundance differences can confound classification results

We find that, in the naive split setting, sex abundance effects play a large role in classification patterns for the non-distinct category of cell types. Within the classification scheme, cell types that are female-dominated in terms of abundance in the original dataset will be sampled more as we increase the overall proportion of female samples in the training set, and vice versa for male-dominated cell types. In turn, this will boost classifier performance for female-dominated cell types on both the male and the female test set, and vice versa for male-dominated cell types. For example, MT-positive alveolar macrophages are female-dominated cell type (Fig. 1(c)); the number of cells in the training set of this type increases as we increase the overall proportion of female cells, yielding a better classifier performance regardless of the test set (Fig. S10(a)). Conversely, we observe the opposite effect in lymphatic endothelial cells (Fig. S10(b)), because the cell type is male-dominated (Fig. 1(c)).

To verify that sex abundance differences can constitute an obscuring factor in classification, we carry out a naive-split classification experiment where we fix the number of cells of one cell type, CD4 T-cells, across all proportions of female cells in the training set (see Methods for details). As a result, its categorization changes from ‘non-distinct’, when its training counts are not fixed, to ‘distinct’ (Fig. S11, Supplementary Table 6), suggesting that differences in abundance across the sampling scheme can indeed obscure classification patterns of individual cell types. However, it should be noted that abundance effects are not absolute — cell types can show distinct patterns in both sex-balanced and differentially abundant cell types (Fig. S12) in the naive classification setting.

In the donor-based classification setting, differences in abundance can also influence observed patterns of classification. When fixing the counts of the two cell types that showed a sex-distinct trend under the donor-based setting, AT0 cells and bronchial goblet cells, the trend only persists for bronchial goblet cells, whereas AT0 cells now also behave non-distinctly (Fig. S13, Supplementary Table 5). This is consistent with the results of our previous matching analysis, where the sex-distinct trend in AT0 cells disappeared after controlling for demographic confounders. Collectively, these findings suggest that classification behavior is vulnerable to multiple sources of confounding even after controlling for donor-level effects, including demographic imbalances and differences in cell type abundance.

## 3 Discussion

In this study, we evaluated the performance of a cell-type classifier separately on male and female cells, under conditions of sex imbalance in the training dataset. If data is split such that the training and test set do not overlap in terms of contributing donors, we find that the classifier does not exacerbate sex bias. Even when trained on highly sex-imbalanced data, there is no significant difference between its performance on male and female cells.

This behavior is apparent both in its overall performance and when we investigate individual cell types: for most cell types, the classifier behaves the same on the male and female test set. When ensuring no donor overlap between train and test set, only two cell types, AT0 cells and bronchial goblet cells, exhibit an increase in performance on female cells and a decrease on male cells as we skew training set proportions towards females. Since these cell types are also significantly differentially abundant between the sexes in the original dataset, a natural hypothesis is that the classifier is incorporating sex-specific information into the learning of cell identities and, as a result, failing to generalize well to the under-represented sex within these two populations. However, after matching donors on relevant covariates and controlling for differences in cell type abundance across training proportions, the apparent sex-distinct trend for AT0 cells is no longer observed, indicating that this effect was largely driven by confounding rather than intrinsic sex-specific signal. For bronchial goblet cells, matching analyses were underpowered, but the trend persists after controlling for abundance, so a contribution of an underlying sex-specific biological signal cannot be excluded. Still, since the majority of cell types do not exhibit sex-biased behavior, it appears that markers of cell identity in scRNA-seq data from the lung can be reliably distinguished by a general-purpose classifier despite sex-based differences in abundance.

To investigate the effect of donor-specific variation, we perform the classification experiment under two settings: with and without taking into account donor information when splitting the data between training and test set. We find that, when a classifier encounters test cells from donors who have contributed to its training set, it performs better than on cells from unseen individuals. This is likely due to the memorization of individual-specific transcriptomic information, across cell types.

As part of the experimental set-up, we vary the proportion of female cells and thus implicitly also the proportion of female donors in the training set; we then aggregate the classification results over sex. Consequently, individual-specific effects can falsely appear sex-specific: the classifier appears to perform better on female cells when trained on female data, but it is in fact simply seeing more female individuals during training leading to inflated accuracy scores on the female test set. Since this apparent sex-biased behavior only occurs when donor overlap exists, we can conclude that it is donor-specific transcriptomic differences that bias the classifier, not sex-specific variation. Data aggregation giving rise to apparent sex-biased patterns has been previously observed when aggregating single-cell expression data to the bulk level if cell population are significantly differentially abundant between the sexes [29]. This underscores the persistent challenge of disentangling sex- and donor-specific effects in gene expression data when the sex distribution is heterogeneous.

Our finding that donor-specific transcriptomic signatures affect classification builds on prior results in the field. Cell type classifiers have been reported to decrease in performance when tested on datasets not seen during training [33], which can be attributed to both technical differences and inter-individual variation. Within-dataset generalization across individuals has been shown to be stable, but larger performance gaps arise if individuals originate from different datasets [34]. Our experiment is conducted on data from a single-cell atlas, composed of distinct datasets but which have been harmonized to mitigate cross-dataset effects; this setting is therefore distinct from both intra- and inter-dataset scenarios previously considered. The decrease of classifier performance on unseen individuals in our case indicates that the memorization of individual-specific transcriptomic features within the learning process persists beyond batch correction for dataset integration. We therefore recommend exercising caution when integrating datasets comprised of few donors into larger single-cell atlases.

Systemic differences in abundance between cell types, resulting from imbalances in the original dataset and compounded by our sampling procedure, represent a confounding factor in the analysis of classification trends. For instance, when donor-based bias is introduced into the experiment by allowing donor overlap between training and test set, abundance effects can negate the influence of individual-specific signatures on classification. Cell types that are heavily male- or female-dominated in abundance will be sampled significantly more or less depending on the sex skew of the training set, leading to identical classification trends on the male and female test set. This abundance-driven behavior occurred in over half of the cell types we investigated in the naive setting, but can be mitigated for a given cell population if sample counts are artificially fixed across training sets.

Our results indicate that differences in cell population sampling, which are very likely in large atlases or cohorts composed of multiple samples collected across different sites by different labs at multiple time points, donor-specific effects can manifest as sex-specific differences. We recommend considering donor differences in the batch correction process and at the same time warrant caution that biological differences across individuals can be lost in the process. Recent developments in learning disentangled representations of single cell data offers an interesting approach to potentially resolve these convoluted signals [50], [51], [52], [53], [54].

There are several limitations of our work which present opportunities for future work. Throughout our investigation, we used the cell type labels as per the HLCA annotation as ground truth. While the batch correction method used to build the atlas is benchmarked for retention of biological variation, integration is performed with the dataset, not the donor, as the batch variable. As a result, donor-level effects may remain uncorrected, introducing bias into the annotation process. Moreover, since some of the constituent datasets of the HLCA are single-sex, sex-based effects may be mistaken as a dataset-level batch effect and removed or distorted.

While this study establishes a proof of concept that cell type classifiers may be affected by inter-individual variation but remain robust to sex distributions in the training set, the classifiers used here are general-purpose. Models that are specific to the scRNA-seq task might behave differently and their internal representation of cell identity may be more susceptible to sex-based variations. For instance, classifiers that employ more advanced feature selection methods, such as label-aware feature space compression [55], clustering of co-expressed genes [56] or manifold preservation based on pairwise cell similarity [57], have been shown to decrease redundancy among selected marker gene sets and filter out overlapping or uninformative signals, which could help mitigate individual-specific noise. Moreover, the classifiers we used are supervised, but unsupervised methods may behave differently under sex- and donor-based bias. On the one hand, unsupervised methods are known to perform worse on data with unbalanced cell type proportions [34], [37]. On the other hand, supervised classifiers are susceptible to reference bias [37], such that, if the reference dataset is biased by individual-level variation, this could exacerbate the decrease in performance observed in unseen donors. Further work should therefore aim to integrate models’ ability to handle sex-skewed data into the benchmarking of automated cell type annotation methods, over a larger variety of models. Additionally, caution is warranted when generalizing these results to other tissues. The lung does not stand out as a particularly sex-dimorphic tissue; future studies performed on tissues that are known to contain cell types with stronger differential expression could yield different results. Moreover, we have only looked at ‘healthy’ lung tissue from the HLCA. Sex differences can manifest differently in diseased tissues [14], [17], [18], [19], [20], [21], [22], [23], which in turn may affect classification performance. Future investigation into other recently-released atlases from the Human Cell Atlas can directly answer these questions.

Individual- and sex-based variation are difficult to decouple, particularly under unbalanced abundance patterns. For instance, a cell type may appear male- or female-dominated when performing a differential abundance analysis on the dataset, but if its samples largely originate from a single donor, markers of cell identity become confounded with both sex-based and donor-specific transcriptomic signatures. Although the HLCA represents the largest effort undergone yet to map the cellular composition of the human lung, certain cell types still exhibit low sample counts or limited donor diversity. At extreme sex ratios or when we filter donors between training and test set, the lack of samples makes it difficult to reliably assess classifier performance. A dataset that is more balanced in terms of cell type distribution, as well as sex distribution within individual cell types, would lend itself to a more complete analysis of performance trends at the individual cell type level, like ours. Furthermore, this entanglement of sex and donor effects is not limited to the classification task, but extends to the cell type labels used for model training and evaluation. We have shown that the original HLCA integration, which corrects batch effects only at the dataset level, may not fully resolve donor-specific transcriptomic variation. As a result, residual donor effects could propagate into the generated embedding and downstream cell type annotation, which would in turn affect our classification experiment. While this issue could in principle be avoided by using data that originates from a single batch, such datasets typically lack the sample size and annotation detail necessary for an analysis like ours. Our batch correction analysis provides a proof of concept that technical artefacts exist in the HLCA integration procedure, but it is limited to a single sub-population. Thus, the extent to which batch correction-induced misattributions occur across the atlas remains unclear. In other cell populations, batch correction may instead obscure genuine biological variation, reflecting over-correction rather than under-correction. Future work should therefore aim to more formally separate technical and biological sources of variation.

Finally, cell type annotation is a multi-class classification problem, where the classifier must distinguish between up to 60 cell types at the finest annotation level. Our experimental set up does not account for the competition between cell types, which may occur especially when cell types are similar to each other in identity (i.e. close-by in the expression space), making them difficult to distinguish by a classifier. In such cases, large abundance variations or sex-dimorphic expression patterns in one cell type may influence performance trends on its competitors. An experimental set-up should thus be devised to evaluate classifier performance independently for each cell type.

Overall, while our results indicate that general-purpose classifiers can distinguish between cell types in the lung even under conditions of sex imbalance, we recommend exercising caution when integrating datasets into single-cell atlases if they are dominated by few donors or exhibit large differences in cell type abundance, as this could confound the cell-type annotation process and give rise to donor-based biases.

## 4 Methods

**Dataset Description.** We used the Human Lung Cell Atlas (HLCA) data [46], sourced from the CELLxGENE database [58]. The atlas is split into the HLCA core, which contains only healthy cells, and the extended HLCA, which contains data across 15 lung diseases; in this paper, we only use the HLCA core. Cell type annotation is hierarchical, over five levels of increasing biological resolution. Here, we only use annotation levels 2–5. Level 1 (which describes major compartments such as ‘Epithelial’, ‘Endothelial’ and ‘Immune’) was excluded, as it represents very coarse distinctions that we considered trivially separable in our classification setting and therefore uninformative for evaluating model performance. Level 2 represents broad lineage groupings within major compartments (e.g. ‘Airway epithelium’, ‘Submucosal gland’, ‘Alveolar epithelium’ are all level 2 labels within the broader level 1 ‘Epithelial’ label), while levels 3–5 provide progressively finer subtype and cell state distinctions (e.g. level 2: ‘Airway epithelium’, level 3: ‘Secretory’, level 4: ‘Club’ and level 5: ‘Club (nasal)’ and ‘Club (non-nasal)’). These levels were defined in a data-driven, recursive manner by cluster subdivision, followed by manual re-annotation by experts in lung biology (see the original HLCA paper [46] for more detail). The data includes both the SCRAN-normalized gene counts and a batch-corrected, 30-dimensional embedding obtained using scANVI [48] during dataset integration. The data is used as an anndata v0.12.1 object [59] in Python for all experiments.

**Differential Abundance Analysis.** Differential abundance analysis was performed using Milo [47] from the pertpy package [60] v1.0.1. Milo builds a KNN graph based on a reduced dimensionality representation of the data — here, we set the scANVI-generated embedding of the HLCA core as the basis of the graph and used k = 30. Subsequently, a set of representative neighborhoods is defined on the KNN graph, followed by counting the number of male and female cells present in each neighborhood and performing differential abundance testing by fitting a negative binomial generalized linear model on the data. Finally, hypothesis testing for differential abundance is performed, while correcting for multiple testing using a weighted FDR procedure that accounts for the spatial overlap of neighborhoods. We use a significance threshold of FDR <0.05 for the differential abundance plot in the main results. A positive log fold-change indicates enrichment for female samples, a negative log fold-change enrichment for male samples. In order to investigate differential abundance in individual cell types, a cell type label is assigned to each neighborhood based on the majority cell type within the neighborhood (where the majority cell type is defined the most frequent cell type label in that neighborhood, at the finest annotation level). This does not lead to significant loss of information as, in most cases, neighborhoods are made up of small sub-populations of the same cell type (Fig. S1(c)); if the fraction of cells in a neighborhood that makes up the majority cell type is below 0.6, that neighborhood is denoted as ‘mixed’ and removed from the subsequent analysis. We use the same threshold of FDR <0.05 to determine significant neighborhoods for individual cell types. To then determine whether a cell type is male- or female-dominated, we compute the median log fold change across its constituent neighborhoods. If the median log fold change is negative, the cell type is considered male-dominated; if it is positive, it is considered female-dominated. Cell types with an absolute median log fold change below 1 are considered sex-balanced.

**Bath correction.** Batch correction of the SMG cell population was performed using Harmony [49] from the harmonypy package v0.2.0. To avoid interference with the prior integration, we use the SCRAN-normalized counts rather than the scANVI embedding. We compute principal components analysis (PCA) with 30 principal components, and run Harmony on the resulted embedding. We apply two separate batch corrections, once using the dataset of origin as the batch variable and once using the donor ID. Briefly, Harmony applies a soft k-means clustering algorithm that penalizes clusters disproportionately populated by cells from a single batch, iteratively adjusting the PCA embedding until convergence. The batch corrected embedding is then used to construct a neighborhood graph (using k-nearest neighbors with 15 neighbors) and subsequently a UMAP representation for visualization. To assess the quality of batch correction, we use the local inverse Simpson’s index (LISI) [49], as implemented with Harmony. LISI scores were computed on the Harmony-corrected embeddings for the integration variable (dataset or donor), cell type and sex. High LISI scores indicate good mixing across batches, while a low LISI score suggests clustering by the batch variable. To visualize the distribution of LISI scores, we use kernel density estimates.

**Classification methods.** Two general-purpose classifiers from the scikit-learn library v1.4.2 [61] were employed for the cell type classification task: K-Nearest Neighbors classifier (KNN) on the embedded data and Random Forest (RF) on the full gene counts, to ensure robustness across data representations. For KNN, the number of neighbors was set to k = 30 for consistency with how the HLCA was built and to match the number of dimensions of the embedding. All other parameters were set to their default values, for both classifiers.

**Experimental set-up.** We employ two settings for the data splitting: naive splitting, where cells are split into training and test set without regard for their donor origin, and donor-based splitting, where we filter donors such that individuals who contribute to the training set do not contribute to the test set. This donor filtering is performed using the GroupShuffleSplit function from the scikit-learn library [61] with the donor ID as the ‘groups’ parameter. Besides this filtering step, the data splitting is performed identically in the two settings, as follows. To ensure a proper balance of classes across the sexes, we first split the data into a male and female subset, then perform train-test splitting within each subset individually. This enables stratification by class within each sex, ensuring that even cell types that are heavily dominated by one sex will have both male and female samples in both the training and the test sets. The male and female test sets are kept fixed and separate throughout the classification experiment, while the individual training sets are merged back together for further processing. To keep the single-sex test sets fixed to the same size, we assign an 80-20 train–test split to the minority sex, while the split for the majority sex is calculated such that the test size equals that of the minority sex. The training set varies across iterations of the classification experiment, to model a gradual variation of the sex imbalance ratio. In total, we generate 11 training sets by varying the proportion of female samples from 0 to 1 in steps of 0.1; this is done by randomly shuffling the training set (to ensure that we do not only sample from a certain part of the set), before sub-setting the indices according to the desired male and female proportions. The classification task is performed at annotation levels 2 through 5. Based on the annotation level, we handle the cell type labels as follows: if a sample does not have a known label at the current level, we iteratively go back through previous annotation levels until a known label is found. As such, the number of classes, and therefore the difficulty of the task, increases with the annotation level - at annotation level 2, the classifier has to discern between 11 classes, whereas, at the finest annotation level, the number of classes is 61. Every classification experiment is repeated four times, over a different random seed.

**Evaluation and performance metrics.** In both data splitting settings, both classifiers are evaluated separately on the male and female test set, for every iteration of the experiment (i.e. for every proportion of female samples in the training set). We compute the average accuracy and the F1 score per class. We also return the normalized confusion matrices for every proportion, to be used for investigating individual classification patterns. For the accuracy plot, we compute the mean and standard deviation across seeds at every proportion. For the F1 score, we determine the median F1 score across seeds for every cell type and at every proportion; thus, every class is represented only once per sex imbalance proportion, but the information across all seeds is captured. Additionally, at every proportion, we determine the statistical significance of the difference between the mean of the F1 score distribution on the male and female test set, using a paired t-test, with significance threshold 0.05. For the donor-based split setting, the unpaired test is used, due to the fact that some classes are missing from either the male or the female test set, rendering the pairing impossible.

**Extraction of classification results on individual cell types.** In order to model behavior on individual cell types, rather than on the entire test set, we make use of the normalized confusion matrices obtained for every proportion of female cells in the training set. The diagonal of the normalized confusion matrix gives, for every cell type, the proportion of correctly classified cells of that type. We consider this to be the model accuracy per cell type and plot it in the same figure for the male and female test set. Next, we want to investigate whether the accuracy on a given cell type is influenced by that cell type’s abundance in the training set. For each cell type, we extract the total number of cells of that type in the training set, as well as how many of these cells are male or female. We plot the male, female and total counts as heat maps accompanying the accuracy per cell type plots. This allows us to investigate whether the counts of a particular cell type change significantly when we vary the overall proportion of female cells in the training set, as well as whether the accuracy of the model appears to be driven by abundance, i.e. increasing as the counts of a cell type increase.

**Categorization of individual cell types.** We categorize cell types as exhibiting a ‘non-distinct’, ‘distinct’ or ‘inconclusive’ trend on the male and female test sets, i.e. does the model perform similarly on male and female cells, better on one of the sexes than the other or is the behavior too erratic to tell. To do this, we define two tests, using the plot of the accuracy per cell type on the male and female test set. First, we calculate the slope of the accuracy curve on the male and female test set and determine whether the two slopes have opposite signs; we call this the ‘slope test’. If this is the case, it indicates that the performance is increasing on sex, but decreasing on the other. Then, we look at whether the accuracy curves on the male and female test sets flip positions in the plot, i.e. if model performance is higher on the female test set when we use the 100% female training set but higher on the male test set when we use the 100% male training set; we call this the ‘flip test’. The reason for using two tests is to check both that performance trends are correlated with the varying proportion of female cells in the training set (slope test), but also that differences on the male and female test set are large enough to be significant (flip test). A cell type is categorized as ‘distinct’ if it fulfills both conditions and ‘non-distinct’ if both tests yield false results. We additionally mark as ‘inconclusive’ all cell types where one of the slopes is close to 0 (below 0.01), reflecting negligible changes in performance across training proportions, or where differences in accuracy are minimal (below 0.042, corresponding to the lower quartile of observed performance differences), indicating little practical separation between sexes. These thresholds are heuristic and intended to exclude qualitatively uninformative patterns rather than to serve as formal statistical tests.

**Verification experiment for naive split setting.** To confirm that the naive data split setting introduces donor-based bias into the classification process, we perform the classification as in the naive setting, but withholding a third of the donors. For two thirds of the donors, train-test splitting, training and evaluation is performed as described above; for the remaining third, only evaluation is performed. Withholding of donors is performed using random sampling without replacement from the pool of donors. Performance is assessed using average accuracy.

**Fixing cell counts.** To assess individual classification patterns independently of abundance patterns, we fix the cell counts for several cell types: CD4 T-cells in the naive setting; suprabasal cells, monocyte-derived macrophages, AT0 cells and bronchial goblet cells in the donor-based setting. To fix the number of cells, we alter the training process such that the sampling procedure is carried out separately on the cell type of interest. Since the sampling function already accounts for a fixed training set size, running it on just one cell type ensures that that population no longer changes in abundance across proportions. This training set is subsequently merged together with the rest of the training samples, then the classification experiment is performed as before.

**Matching analysis.** To control for potential confounding by demographic covariates, male and female donors were matched on smoking status and tissue processing site. Matching was performed greedily: for each male donor, the first available female donor with identical values for both smoking status and tissue processing site was selected, and matched donors were subsequently removed from the pool to prevent reassignment. The resulting dataset of matched donor pairs was then used as input to the classification experiment under the donor-based split, as in the non-matched main experiment.

## CRediT authorship contribution statement

**Nora Ghenciulescu:** Designed the experiments, Performed all the experiments and prepared all the figures, Wrote the main manuscript text. **Marcel J. Reinders:** Conceived the study, Reviewed and edited the manuscript text. **Ahmed Mahfouz:** Conceived the study, Designed the experiments, Reviewed and edited the manuscript text.

## Code availability

The code used for results and figures is available at https://github.com/noraghenciulescu01/Sex-donor-bias-in-cell-type-classification-scRNAseq. Research reported in this work was partially facilitated by computational resources and support of the Delft AI Cluster (DAIC) at TU Delft (https://doc.daic.tudelft.nl). All analyses were performed in Python v3.12.3.

## Funding

This research did not receive funding.

## Declaration of competing interest

The authors declare that they have no known competing financial interests or personal relationships that could have appeared to influence the work reported in this paper.

## Data Availability

All data used is already publicly available and our code is openly public on GitHub as indicated in the data availability section.

## Appendix A Supplementary data

MMC S1
