# Supplementary material for: Donor-specific, but not sex-specific, signatures dominate cell-type classification in lung scRNA-seq data
Source: Biochem Biophys Rep. 2026 Jul 17;47:102692. doi: 10.1016/j.bbrep.2026.102692 (PMC13400325; doi:10.1016/j.bbrep.2026.102692)
Supplement: MMC S1 [file mmc1.pdf]

Supplementary materials

Supplementary Table 1: Summary of results from the differential abundance analysis. Per cell type: the median log Fold Change (Female/Male), total number of neighborhoods, number of neighborhoods significantly enriched for female samples, number of neighborhoods significantly enriched for male samples. Negative log Fold Change indicates enrichment for male samples, positive indicates enrichment for female samples. Cell types are considered 'sex-balanced' if their absolute log Fold change is below 1. Cell types are ordered as in Figure 1c.

Cell type	Median logFC	Total nhoods	Signif. female nhoods	Signif. male nhoods	Enrichment
Goblet (bronchial)	-3.982	106	13	41	Male-dominated
Mesothelium	-3.335	17	0	8	Male-dominated
Adventitial fibroblasts	-2.696	781	13	137	Male-dominated
Alveolar macrophages	-2.100	4728	630	682	Male-dominated
Alveolar Mph CCL3+	-2.073	556	10	21	Male-dominated
EC aerocyte capillary	-1.982	507	33	8	Male-dominated
Lymphatic EC differentiating	-1.914	33	0	0	Male-dominated
Pericytes	-1.894	192	2	0	Male-dominated
Monocyte-derived Mph	-1.886	1927	42	179	Male-dominated
Interstitial Mph perivascular	-1.877	298	0	13	Male-dominated
EC general capillary	-1.783	1090	33	30	Male-dominated
Subpleural fibroblasts	-1.706	16	4	0	Male-dominated
EC arterial	-1.668	508	3	12	Male-dominated
pre-TB secretory	-1.661	284	2	2	Male-dominated
SMG serous (bronchial)	-1.499	99	0	0	Male-dominated
EC venous pulmonary	-1.487	459	0	16	Male-dominated
Non-classical monocytes	-1.373	614	5	20	Male-dominated
Lymphatic EC mature	-1.318	259	0	3	Male-dominated
SM activated stress response	-1.112	44	2	0	Male-dominated
Multiciliated (nasal)	-1.060	325	59	15	Male-dominated
Peribronchial fibroblasts	-0.993	110	3	0	Balanced
Alveolar Mph proliferating	-0.984	65	1	0	Balanced
Goblet (subsegmental)	-0.920	62	1	1	Balanced
Plasmacytoid DCs	-0.747	39	0	0	Balanced
CD8 T cells	-0.693	1992	142	35	Balanced
Ionocyte	-0.679	44	0	0	Balanced
B cells	-0.635	297	3	1	Balanced
Neuroendocrine	-0.496	9	0	0	Balanced
Multiciliated (non-nasal)	-0.486	2521	62	69	Balanced
AT1	-0.461	564	30	1	Balanced
SMG mucous	-0.346	41	2	0	Balanced
Alveolar fibroblasts	-0.266	386	38	1	Balanced
Lymphatic EC proliferating	-0.235	1	0	0	Balanced
Basal resting	-0.215	2703	177	206	Balanced
DC2	-0.174	607	6	8	Balanced
Club (nasal)	0.080	1789	217	39	Balanced
EC venous systemic	0.196	457	21	5	Balanced
Classical monocytes	0.295	1237	117	8	Balanced
SMG duct	0.327	85	0	0	Balanced
Suprabasal	0.472	2820	509	239	Balanced
Smooth muscle	0.511	219	4	0	Balanced
Migratory DCs	0.511	20	1	0	Balanced
AT2	0.546	4163	1117	415	Balanced
Myofibroblasts	0.574	44	2	0	Balanced
Club (non-nasal)	0.671	655	50	28	Balanced
Plasma cells	0.716	135	4	1	Balanced
DC1	0.717	16	0	0	Balanced
Goblet (nasal)	0.718	2485	306	102	Balanced
Deuterosomal	0.768	72	0	0	Balanced
Hematopoietic stem cells	0.974	2	0	0	Balanced
Tuft	1.233	10	0	0	Female-dominated
CD4 T cells	1.315	1410	156	6	Female-dominated
Hillock-like	1.497	295	24	29	Female-dominated
NK cells	1.653	1129	105	0	Female-dominated
Smooth muscle FAM83D+	1.849	15	0	0	Female-dominated
Mast cells	1.862	417	65	1	Female-dominated
AT2 proliferating	2.357	68	7	0	Female-dominated
T cells proliferating	2.420	20	3	0	Female-dominated
AT0	2.882	86	16	0	Female-dominated
Alveolar Mph MT-positive	2.917	71	14	0	Female-dominated
SMG serous (nasal)	3.852	106	58	0	Female-dominated

Supplementary Table 2: Donor overlap between train and test sets in the naive split setting. Rows correspond to individual donors, columns correspond to the proportion of female cells in the training set. Each cell is formatted as train counts/test counts, with the computed ratio shown in parentheses as a measure of overlap. Donor IDs have been shortened from the original in the HLCA data by removing the common prefix. All donors overlap between training and test set, except in the cases where the train set is 0% female or 100% female. For female donors, the train-to-test ratio increases as we increase the proportion of female cells in the training set, and vice versa for male donors. This corresponds to the increase in classification performance on the female test set and decrease in performance on the male test set in the naive setting experiment (Figure 3c), indicating that the apparent sex-biased classification effect is driven by memorization of individual-specific features due to donor leakage between train and test set. The only exception is male donor 'VUHD87', who only contributed 8 cells to the overall HLCA core and is therefore almost entirely left out of the training and testing under all proportions.

**Donor**	**0.0**	**0.1**	**0.2**	**0.3**	**0.4**	**0.5**	**0.6**	**0.7**	**0.8**	**0.9**	**1.0**
**Female donors (train counts / test counts)**											
2020-3173-NC004	0/2340 (0.00)	923/2340 (0.39)	1905/2340 (0.81)	2881/2340 (1.23)	3815/2340 (1.63)	4710/2340 (2.01)	5607/2340 (2.40)	6524/2340 (2.79)	7520/2340 (3.21)	8453/2340 (3.61)	9385/2340 (4.01)
2020-3173-NC005	0/1255 (0.00)	516/1255 (0.41)	1023/1255 (0.82)	1553/1255 (1.24)	2065/1255 (1.65)	2550/1255 (2.03)	3053/1255 (2.43)	3536/1255 (2.82)	3997/1255 (3.18)	4480/1255 (3.57)	5000/1255 (3.98)
2020-3173-NC007	0/649 (0.00)	268/649 (0.41)	572/649 (0.88)	866/649 (1.33)	1153/649 (1.78)	1406/649 (2.17)	1686/649 (2.60)	1939/649 (2.99)	2206/649 (3.40)	2498/649 (3.85)	2778/649 (4.28)
290B	0/432 (0.00)	156/432 (0.36)	306/432 (0.71)	474/432 (1.10)	634/432 (1.47)	803/432 (1.86)	941/432 (2.18)	1111/432 (2.57)	1253/432 (2.90)	1413/432 (3.27)	1559/432 (3.61)
296C	0/732 (0.00)	277/732 (0.38)	617/732 (0.84)	887/732 (1.21)	1182/732 (1.61)	1534/732 (2.10)	1794/732 (2.45)	2107/732 (2.88)	2443/732 (3.34)	2723/732 (3.72)	3039/732 (4.15)
356C	0/2693 (0.00)	1155/2693 (0.43)	2270/2693 (0.84)	3326/2693 (1.24)	4386/2693 (1.63)	5481/2693 (2.04)	6564/2693 (2.44)	7661/2693 (2.84)	8699/2693 (3.23)	9810/2693 (3.64)	10864/2693 (4.03)
390C	0/2936 (0.00)	1152/2936 (0.39)	2367/2936 (0.81)	3562/2936 (1.21)	4735/2936 (1.61)	5867/2936 (2.00)	7067/2936 (2.41)	8294/2936 (2.82)	9485/2936 (3.23)	10646/2936 (3.63)	11792/2936 (4.02)
D322	0/751 (0.00)	287/751 (0.38)	582/751 (0.77)	851/751 (1.13)	1144/751 (1.52)	1455/751 (1.94)	1753/751 (2.33)	2031/751 (2.70)	2324/751 (3.09)	2592/751 (3.45)	2877/751 (3.83)
D337	0/259 (0.00)	111/259 (0.43)	236/259 (0.91)	345/259 (1.33)	469/259 (1.81)	586/259 (2.26)	707/259 (2.73)	810/259 (3.13)	917/259 (3.54)	1020/259 (3.94)	1117/259 (4.31)
D344	0/1255 (0.00)	480/1255 (0.38)	937/1255 (0.75)	1371/1255 (1.09)	1879/1255 (1.50)	2340/1255 (1.86)	2803/1255 (2.23)	3280/1255 (2.61)	3769/1255 (3.00)	4260/1255 (3.39)	4746/1255 (3.78)
D353	0/3074 (0.00)	1265/3074 (0.41)	2431/3074 (0.79)	3652/3074 (1.19)	4863/3074 (1.58)	6121/3074 (1.99)	7342/3074 (2.39)	8595/3074 (2.80)	9778/3074 (3.18)	11059/3074 (3.60)	12306/3074 (4.00)
D363	0/1517 (0.00)	573/1517 (0.38)	1204/1517 (0.79)	1802/1517 (1.19)	2390/1517 (1.58)	3018/1517 (1.99)	3632/1517 (2.39)	4223/1517 (2.78)	4840/1517 (3.19)	5438/1517 (3.58)	6026/1517 (3.97)
D372	0/2631 (0.00)	1068/2631 (0.41)	2168/2631 (0.82)	3254/2631 (1.24)	4313/2631 (1.64)	5389/2631 (2.05)	6425/2631 (2.44)	7472/2631 (2.84)	8486/2631 (3.23)	9586/2631 (3.64)	10633/2631 (4.04)
Donor_03	0/1029 (0.00)	422/1029 (0.41)	832/1029 (0.81)	1241/1029 (1.21)	1650/1029 (1.60)	2085/1029 (2.03)	2521/1029 (2.45)	2925/1029 (2.84)	3301/1029 (3.21)	3697/1029 (3.59)	4134/1029 (4.02)
Donor_04	0/1051 (0.00)	427/1051 (0.41)	793/1051 (0.75)	1216/1051 (1.16)	1624/1051 (1.55)	2057/1051 (1.96)	2438/1051 (2.32)	2834/1051 (2.70)	3253/1051 (3.10)	3671/1051 (3.49)	4113/1051 (3.91)
Donor_05	0/1043 (0.00)	409/1043 (0.39)	815/1043 (0.78)	1249/1043 (1.20)	1696/1043 (1.63)	2064/1043 (1.98)	2513/1043 (2.41)	2954/1043 (2.83)	3376/1043 (3.24)	3799/1043 (3.64)	4239/1043 (4.06)
Donor_06	0/858 (0.00)	325/858 (0.38)	637/858 (0.74)	960/858 (1.12)	1306/858 (1.52)	1660/858 (1.93)	1998/858 (2.33)	2393/858 (2.79)	2724/858 (3.17)	3051/858 (3.56)	3383/858 (3.94)
Donor_07	0/1466 (0.00)	558/1466 (0.38)	1151/1466 (0.79)	1781/1466 (1.21)	2331/1466 (1.59)	2925/1466 (2.00)	3516/1466 (2.40)	4085/1466 (2.79)	4615/1466 (3.15)	5206/1466 (3.55)	5802/1466 (3.96)
GRO-02	0/182 (0.00)	67/182 (0.37)	144/182 (0.79)	219/182 (1.20)	305/182 (1.68)	377/182 (2.07)	463/182 (2.54)	537/182 (2.95)	610/182 (3.35)	681/182 (3.74)	760/182 (4.18)
GRO-09	0/3257 (0.00)	1284/3257 (0.39)	2564/3257 (0.79)	3804/3257 (1.17)	5109/3257 (1.57)	6373/3257 (1.96)	7697/3257 (2.36)	8975/3257 (2.76)	10327/3257 (3.17)	11633/3257 (3.57)	12930/3257 (3.97)
T120	0/340 (0.00)	129/340 (0.38)	259/340 (0.76)	372/340 (1.09)	535/340 (1.57)	686/340 (2.02)	860/340 (2.53)	992/340 (2.92)	1160/340 (3.41)	1306/340 (3.84)	1446/340 (4.25)
T121	0/265 (0.00)	99/265 (0.37)	191/265 (0.72)	306/265 (1.15)	435/265 (1.64)	567/265 (2.14)	688/265 (2.60)	814/265 (3.07)	932/265 (3.52)	1026/265 (3.87)	1154/265 (4.35)
T126	0/347 (0.00)	138/347 (0.40)	294/347 (0.85)	442/347 (1.27)	565/347 (1.63)	704/347 (2.03)	854/347 (2.46)	996/347 (2.87)	1148/347 (3.31)	1276/347 (3.68)	1428/347 (4.12)
T137	0/292 (0.00)	107/292 (0.37)	205/292 (0.70)	304/292 (1.04)	405/292 (1.39)	505/292 (1.73)	608/292 (2.08)	713/292 (2.44)	829/292 (2.84)	940/292 (3.22)	1040/292 (3.56)
T166	0/541 (0.00)	195/541 (0.36)	410/541 (0.76)	609/541 (1.13)	799/541 (1.48)	1022/541 (1.89)	1240/541 (2.29)	1444/541 (2.67)	1663/541 (3.07)	1875/541 (3.47)	2085/541 (3.85)
T167	0/553 (0.00)	241/553 (0.44)	471/553 (0.85)	695/553 (1.26)	941/553 (1.70)	1172/553 (2.12)	1391/553 (2.52)	1638/553 (2.96)	1880/553 (3.40)	2092/553 (3.78)	2314/553 (4.18)
T89	0/431 (0.00)	180/431 (0.42)	347/431 (0.81)	534/431 (1.24)	708/431 (1.64)	872/431 (2.02)	1070/431 (2.48)	1248/431 (2.90)	1454/431 (3.37)	1610/431 (3.74)	1801/431 (4.18)
THD0009	0/317 (0.00)	148/317 (0.47)	288/317 (0.91)	432/317 (1.36)	598/317 (1.89)	733/317 (2.31)	889/317 (2.80)	1020/317 (3.22)	1143/317 (3.61)	1276/317 (4.03)	1422/317 (4.49)
VUHD072	0/118 (0.00)	40/118 (0.34)	75/118 (0.64)	126/118 (1.07)	184/118 (1.56)	229/118 (1.94)	281/118 (2.38)	322/118 (2.73)	384/118 (3.25)	439/118 (3.72)	483/118 (4.09)
VUHD073	0/52 (0.00)	26/52 (0.50)	51/52 (0.98)	79/52 (1.52)	105/52 (2.02)	128/52 (2.46)	147/52 (2.83)	161/52 (3.10)	185/52 (3.56)	201/52 (3.87)	226/52 (4.35)
VUHD077	0/85 (0.00)	22/85 (0.26)	48/85 (0.56)	90/85 (1.06)	129/85 (1.52)	156/85 (1.84)	193/85 (2.27)	220/85 (2.59)	250/85 (2.94)	270/85 (3.18)	292/85 (3.44)
VUHD67	0/1954 (0.00)	833/1954 (0.43)	1645/1954 (0.84)	2485/1954 (1.27)	3278/1954 (1.68)	4072/1954 (2.08)	4863/1954 (2.49)	5696/1954 (2.92)	6519/1954 (3.34)	7310/1954 (3.74)	8139/1954 (4.17)

Continued on next page

Donor	0.0	0.1	0.2	0.3	0.4	0.5	0.6	0.7	0.8	0.9	1.0
VUHD69	0/243 (0.00)	78/243 (0.32)	181/243 (0.74)	276/243 (1.14)	375/243 (1.54)	467/243 (1.92)	577/243 (2.37)	662/243 (2.72)	765/243 (3.15)	857/243 (3.53)	965/243 (3.97)
VUHD84	0/1862 (0.00)	695/1862 (0.37)	1431/1862 (0.77)	2141/1862 (1.15)	2882/1862 (1.55)	3643/1862 (1.96)	4385/1862 (2.35)	5131/1862 (2.76)	5832/1862 (3.13)	6525/1862 (3.50)	7272/1862 (3.91)
VUHD95	0/1517 (0.00)	630/1517 (0.42)	1214/1517 (0.80)	1798/1517 (1.19)	2415/1517 (1.59)	3015/1517 (1.99)	3647/1517 (2.40)	4245/1517 (2.80)	4834/1517 (3.19)	5459/1517 (3.60)	6043/1517 (3.98)
donor 3	0/5016 (0.00)	2042/5016 (0.41)	4004/5016 (0.80)	5976/5016 (1.19)	7870/5016 (1.57)	9839/5016 (1.96)	11780/5016 (2.35)	13743/5016 (2.74)	15732/5016 (3.14)	17711/5016 (3.53)	19651/5016 (3.92)
lafy_donor_4	0/885 (0.00)	341/885 (0.39)	682/885 (0.77)	1037/885 (1.17)	1388/885 (1.57)	1722/885 (1.95)	2076/885 (2.35)	2425/885 (2.74)	2795/885 (3.16)	3175/885 (3.59)	3546/885 (4.01)
lafy_donor_6	0/2010 (0.00)	828/2010 (0.41)	1640/2010 (0.82)	2489/2010 (1.24)	3319/2010 (1.65)	4143/2010 (2.06)	4902/2010 (2.44)	5710/2010 (2.84)	6533/2010 (3.25)	7392/2010 (3.68)	8162/2010 (4.06)
**Male donors (train counts / test counts)**											
2020-3173 -NC002	2544/608 (4.18)	2286/608 (3.76)	2037/608 (3.35)	1769/608 (2.91)	1517/608 (2.50)	1252/608 (2.06)	1022/608 (1.68)	776/608 (1.28)	530/608 (0.87)	244/608 (0.40)	0/608 (0.00)
2020-3173 -NC003	1636/403 (4.06)	1466/403 (3.64)	1310/403 (3.25)	1147/403 (2.85)	976/403 (2.42)	827/403 (2.05)	660/403 (1.64)	476/403 (1.18)	323/403 (0.80)	165/403 (0.41)	0/403 (0.00)
2020-3173 -NC006	4488/1174 (3.82)	4055/1174 (3.45)	3597/1174 (3.06)	3112/1174 (2.65)	2632/1174 (2.24)	2215/1174 (1.89)	1733/1174 (1.48)	1301/1174 (1.11)	873/1174 (0.74)	448/1174 (0.38)	0/1174 (0.00)
2020-3173 -NC008	3945/1012 (3.90)	3570/1012 (3.53)	3175/1012 (3.14)	2772/1012 (2.74)	2393/1012 (2.36)	2009/1012 (1.99)	1587/1012 (1.57)	1190/1012 (1.18)	760/1012 (0.75)	363/1012 (0.36)	0/1012 (0.00)
284C	183/47 (3.89)	166/47 (3.53)	147/47 (3.13)	131/47 (2.79)	112/47 (2.38)	92/47 (1.96)	72/47 (1.53)	55/47 (1.17)	34/47 (0.72)	15/47 (0.32)	0/47 (0.00)
292B	187/51 (3.67)	169/51 (3.31)	149/51 (2.92)	123/51 (2.41)	107/51 (2.10)	84/51 (1.65)	68/51 (1.33)	53/51 (1.04)	33/51 (0.65)	14/51 (0.27)	0/51 (0.00)
298C	1283/293 (4.38)	1155/293 (3.94)	1022/293 (3.49)	893/293 (3.05)	761/293 (2.60)	639/293 (2.18)	496/293 (1.69)	391/293 (1.33)	268/293 (0.91)	139/293 (0.47)	0/293 (0.00)
302C	1746/407 (4.29)	1585/407 (3.89)	1397/407 (3.43)	1225/407 (3.01)	1046/407 (2.57)	870/407 (2.14)	714/407 (1.75)	530/407 (1.30)	333/407 (0.82)	174/407 (0.43)	0/407 (0.00)
368C	3809/921 (4.14)	3421/921 (3.71)	3027/921 (3.29)	2634/921 (2.86)	2262/921 (2.46)	1876/921 (2.04)	1497/921 (1.63)	1127/921 (1.22)	725/921 (0.79)	365/921 (0.40)	0/921 (0.00)
D326	2659/715 (3.72)	2360/715 (3.30)	2126/715 (2.97)	1876/715 (2.62)	1596/715 (2.23)	1363/715 (1.91)	1092/715 (1.53)	812/715 (1.14)	524/715 (0.73)	257/715 (0.36)	0/715 (0.00)
D339	3069/761 (4.03)	2747/761 (3.61)	2415/761 (3.17)	2112/761 (2.78)	1813/761 (2.38)	1520/761 (2.00)	1207/761 (1.59)	888/761 (1.17)	594/761 (0.78)	312/761 (0.41)	0/761 (0.00)
D354	3532/894 (3.95)	3183/894 (3.56)	2819/894 (3.15)	2455/894 (2.75)	2151/894 (2.41)	1805/894 (2.02)	1440/894 (1.61)	1070/894 (1.20)	693/894 (0.78)	353/894 (0.39)	0/894 (0.00)
D367	4980/1248 (3.99)	4492/1248 (3.60)	3976/1248 (3.19)	3501/1248 (2.81)	3023/1248 (2.42)	2529/1248 (2.03)	2024/1248 (1.62)	1504/1248 (1.21)	1013/1248 (0.81)	500/1248 (0.40)	0/1248 (0.00)
Donor_01	2842/719 (3.95)	2572/719 (3.58)	2280/719 (3.17)	2004/719 (2.79)	1726/719 (2.40)	1416/719 (1.97)	1151/719 (1.60)	849/719 (1.18)	563/719 (0.78)	281/719 (0.39)	0/719 (0.00)
Donor_02	1932/508 (3.80)	1723/508 (3.39)	1545/508 (3.04)	1342/508 (2.64)	1143/508 (2.25)	938/508 (1.85)	747/508 (1.47)	547/508 (1.08)	351/508 (0.69)	180/508 (0.35)	0/508 (0.00)
Donor_08	2536/632 (4.01)	2285/632 (3.62)	2036/632 (3.22)	1808/632 (2.86)	1544/632 (2.44)	1279/632 (2.02)	1015/632 (1.61)	760/632 (1.20)	505/632 (0.80)	252/632 (0.40)	0/632 (0.00)
GRO-01	478/116 (4.12)	426/116 (3.67)	379/116 (3.27)	328/116 (2.83)	273/116 (2.35)	219/116 (1.89)	177/116 (1.53)	135/116 (1.16)	95/116 (0.82)	57/116 (0.49)	0/116 (0.00)
GRO-03	6596/1690 (3.90)	5886/1690 (3.48)	5262/1690 (3.11)	4578/1690 (2.71)	3924/1690 (2.32)	3268/1690 (1.93)	2632/1690 (1.56)	1998/1690 (1.18)	1316/1690 (0.78)	643/1690 (0.38)	0/1690 (0.00)
GRO-04	6997/1777 (3.94)	6313/1777 (3.55)	5654/1777 (3.18)	4913/1777 (2.76)	4214/1777 (2.37)	3487/1777 (1.96)	2759/1777 (1.55)	2060/1777 (1.16)	1362/1777 (0.77)	674/1777 (0.38)	0/1777 (0.00)
GRO-06	1880/458 (4.10)	1709/458 (3.73)	1523/458 (3.33)	1340/458 (2.93)	1150/458 (2.51)	952/458 (2.08)	763/458 (1.67)	561/458 (1.22)	380/458 (0.83)	190/458 (0.41)	0/458 (0.00)
GRO-07	2409/601 (4.01)	2180/601 (3.63)	1919/601 (3.19)	1685/601 (2.80)	1468/601 (2.44)	1222/601 (2.03)	1005/601 (1.67)	760/601 (1.26)	513/601 (0.85)	266/601 (0.44)	0/601 (0.00)
GRO-08	1605/388 (4.14)	1457/388 (3.76)	1283/388 (3.31)	1109/388 (2.86)	952/388 (2.45)	769/388 (1.98)	624/388 (1.61)	466/388 (1.20)	310/388 (0.80)	151/388 (0.39)	0/388 (0.00)
GRO-10	6682/1637 (4.08)	6033/1637 (3.69)	5346/1637 (3.27)	4657/1637 (2.84)	4006/1637 (2.45)	3323/1637 (2.03)	2631/1637 (1.61)	1984/1637 (1.21)	1374/1637 (0.84)	701/1637 (0.43)	0/1637 (0.00)
GRO-11	1114/294 (3.79)	1011/294 (3.44)	904/294 (3.07)	788/294 (2.68)	682/294 (2.32)	585/294 (1.99)	450/294 (1.53)	346/294 (1.18)	244/294 (0.83)	116/294 (0.39)	0/294 (0.00)
NU_CZI01	14746/3679 (4.01)	13265/3679 (3.61)	11843/3679 (3.22)	10365/3679 (2.82)	8833/3679 (2.40)	7360/3679 (2.00)	5862/3679 (1.59)	4405/3679 (1.20)	2897/3679 (0.79)	1424/3679 (0.39)	0/3679 (0.00)
NU_CZI02	19175/4862 (3.94)	17313/4862 (3.56)	15348/4862 (3.16)	13441/4862 (2.76)	11610/4862 (2.39)	9714/4862 (2.00)	7800/4862 (1.60)	5908/4862 (1.22)	3929/4862 (0.81)	1959/4862 (0.40)	0/4862 (0.00)
T101	1758/403 (4.36)	1583/403 (3.93)	1402/403 (3.48)	1235/403 (3.06)	1048/403 (2.60)	887/403 (2.20)	699/403 (1.73)	537/403 (1.33)	367/403 (0.91)	212/403 (0.53)	0/403 (0.00)
T153	1843/401 (4.60)	1636/401 (4.08)	1441/401 (3.59)	1268/401 (3.16)	1068/401 (2.66)	886/401 (2.21)	704/401 (1.76)	510/401 (1.27)	337/401 (0.84)	150/401 (0.37)	0/401 (0.00)
T154	849/224 (3.79)	761/224 (3.40)	688/224 (3.07)	594/224 (2.65)	519/224 (2.32)	440/224 (1.96)	365/224 (1.63)	276/224 (1.23)	207/224 (0.92)	103/224 (0.46)	0/224 (0.00)
T164	1747/420 (4.16)	1567/420 (3.73)	1402/420 (3.34)	1218/420 (2.90)	1042/420 (2.48)	864/420 (2.06)	713/420 (1.70)	520/420 (1.24)	327/420 (0.78)	163/420 (0.39)	0/420 (0.00)
T165	1114/257 (4.33)	1018/257 (3.96)	921/257 (3.58)	808/257 (3.14)	685/257 (2.67)	569/257 (2.21)	451/257 (1.75)	329/257 (1.28)	219/257 (0.85)	106/257 (0.41)	0/257 (0.00)
T85	1827/523 (3.49)	1629/523 (3.11)	1438/523 (2.75)	1269/523 (2.43)	1094/523 (2.09)	913/523 (1.75)	745/523 (1.42)	540/523 (1.03)	373/523 (0.71)	178/523 (0.34)	0/523 (0.00)
T90	1177/275 (4.28)	1082/275 (3.93)	949/275 (3.45)	835/275 (3.04)	708/275 (2.57)	595/275 (2.16)	471/275 (1.71)	346/275 (1.26)	240/275 (0.87)	127/275 (0.46)	0/275 (0.00)
THD0001	1317/335 (3.93)	1184/335 (3.53)	1076/335 (3.21)	943/335 (2.81)	793/335 (2.37)	648/335 (1.93)	526/335 (1.57)	405/335 (1.21)	289/335 (0.86)	150/335 (0.45)	0/335 (0.00)

Continued on next page

Donor	0.0	0.1	0.2	0.3	0.4	0.5	0.6	0.7	0.8	0.9	1.0
THD0005	2381/579 (4.11)	2150/579 (3.71)	1904/579 (3.29)	1680/579 (2.90)	1414/579 (2.44)	1194/579 (2.06)	935/579 (1.61)	691/579 (1.19)	459/579 (0.79)	220/579 (0.38)	0/579 (0.00)
THD0006	675/177 (3.81)	615/177 (3.47)	551/177 (3.11)	488/177 (2.76)	415/177 (2.34)	343/177 (1.94)	285/177 (1.61)	208/177 (1.18)	146/177 (0.82)	75/177 (0.42)	0/177 (0.00)
THD0007	2186/560 (3.90)	1963/560 (3.51)	1752/560 (3.13)	1547/560 (2.76)	1318/560 (2.35)	1105/560 (1.97)	893/560 (1.59)	657/560 (1.17)	445/560 (0.79)	223/560 (0.40)	0/560 (0.00)
THD0008	1637/446 (3.67)	1490/446 (3.34)	1355/446 (3.04)	1191/446 (2.67)	1017/446 (2.28)	865/446 (1.94)	681/446 (1.53)	540/446 (1.21)	336/446 (0.75)	155/446 (0.35)	0/446 (0.00)
THD0010	399/91 (4.38)	370/91 (4.07)	330/91 (3.63)	281/91 (3.09)	240/91 (2.64)	196/91 (2.15)	161/91 (1.77)	117/91 (1.29)	78/91 (0.86)	36/91 (0.40)	0/91 (0.00)
THD0012	527/104 (5.07)	469/104 (4.51)	422/104 (4.06)	357/104 (3.43)	309/104 (2.97)	249/104 (2.39)	206/104 (1.98)	149/104 (1.43)	107/104 (1.03)	52/104 (0.50)	0/104 (0.00)
THD0014	1007/256 (3.93)	905/256 (3.54)	813/256 (3.18)	711/256 (2.78)	599/256 (2.34)	493/256 (1.93)	412/256 (1.61)	312/256 (1.22)	204/256 (0.80)	98/256 (0.38)	0/256 (0.00)
THD0015	780/193 (4.04)	707/193 (3.66)	623/193 (3.23)	546/193 (2.83)	476/193 (2.47)	403/193 (2.09)	326/193 (1.69)	242/193 (1.25)	159/193 (0.82)	87/193 (0.45)	0/193 (0.00)
THD0017	710/181 (3.92)	633/181 (3.50)	553/181 (3.06)	494/181 (2.73)	429/181 (2.37)	349/181 (1.93)	270/181 (1.49)	206/181 (1.14)	144/181 (0.80)	73/181 (0.40)	0/181 (0.00)
THD0019	875/263 (3.33)	779/263 (2.96)	690/263 (2.62)	623/263 (2.37)	544/263 (2.07)	448/263 (1.70)	367/263 (1.40)	269/263 (1.02)	176/263 (0.67)	87/263 (0.33)	0/263 (0.00)
THD0020	395/114 (3.46)	352/114 (3.09)	310/114 (2.72)	274/114 (2.40)	228/114 (2.00)	185/114 (1.62)	147/114 (1.29)	115/114 (1.01)	82/114 (0.72)	42/114 (0.37)	0/114 (0.00)
VUHD071	3148/765 (4.12)	2805/765 (3.67)	2467/765 (3.22)	2172/765 (2.84)	1802/765 (2.36)	1457/765 (1.90)	1166/765 (1.52)	920/765 (1.20)	602/765 (0.79)	295/765 (0.39)	0/765 (0.00)
VUHD076	24/6 (4.00)	20/6 (3.33)	16/6 (2.67)	15/6 (2.50)	10/6 (1.67)	7/6 (1.17)	6/6 (1.00)	5/6 (0.83)	5/6 (0.83)	1/6 (0.17)	0/6 (0.00)
VUHD078	247/49 (5.04)	220/49 (4.49)	193/49 (3.94)	168/49 (3.43)	147/49 (3.00)	123/49 (2.51)	99/49 (2.02)	75/49 (1.53)	49/49 (1.00)	22/49 (0.45)	0/49 (0.00)
VUHD080	226/54 (4.19)	199/54 (3.69)	174/54 (3.22)	143/54 (2.65)	117/54 (2.17)	100/54 (1.85)	83/54 (1.54)	70/54 (1.30)	47/54 (0.87)	26/54 (0.48)	0/54 (0.00)
VUHD101	1627/415 (3.92)	1460/415 (3.52)	1285/415 (3.10)	1129/415 (2.72)	970/415 (2.34)	813/415 (1.96)	648/415 (1.56)	487/415 (1.17)	320/415 (0.77)	165/415 (0.40)	0/415 (0.00)
VUHD103	5007/1216 (4.12)	4488/1216 (3.69)	3965/1216 (3.26)	3442/1216 (2.83)	2992/1216 (2.46)	2502/1216 (2.06)	2003/1216 (1.65)	1503/1216 (1.24)	995/1216 (0.82)	494/1216 (0.41)	0/1216 (0.00)
VUHD104	3291/777 (4.24)	2966/777 (3.82)	2616/777 (3.37)	2301/777 (2.96)	1976/777 (2.54)	1660/777 (2.14)	1335/777 (1.72)	1037/777 (1.33)	699/777 (0.90)	368/777 (0.47)	0/777 (0.00)
VUHD105	88/14 (6.29)	74/14 (5.29)	66/14 (4.71)	59/14 (4.21)	53/14 (3.79)	44/14 (3.14)	39/14 (2.79)	33/14 (2.36)	21/14 (1.50)	13/14 (0.93)	0/14 (0.00)
VUHD106	4865/1232 (3.95)	4381/1232 (3.56)	3889/1232 (3.16)	3399/1232 (2.76)	2906/1232 (2.36)	2437/1232 (1.98)	1949/1232 (1.58)	1470/1232 (1.19)	969/1232 (0.79)	488/1232 (0.40)	0/1232 (0.00)
VUHD107	519/150 (3.46)	478/150 (3.19)	411/150 (2.74)	367/150 (2.45)	320/150 (2.13)	264/150 (1.76)	209/150 (1.39)	155/150 (1.03)	96/150 (0.64)	40/150 (0.27)	0/150 (0.00)
VUHD66	1815/468 (3.88)	1640/468 (3.50)	1439/468 (3.07)	1281/468 (2.74)	1064/468 (2.27)	868/468 (1.85)	701/468 (1.50)	526/468 (1.12)	340/468 (0.73)	162/468 (0.35)	0/468 (0.00)
VUHD68	4067/1011 (4.02)	3660/1011 (3.62)	3248/1011 (3.21)	2853/1011 (2.82)	2487/1011 (2.46)	2057/1011 (2.03)	1622/1011 (1.60)	1217/1011 (1.20)	811/1011 (0.80)	412/1011 (0.41)	0/1011 (0.00)
VUHD70	1171/318 (3.68)	1046/318 (3.29)	919/318 (2.89)	809/318 (2.54)	694/318 (2.18)	580/318 (1.82)	446/318 (1.40)	348/318 (1.09)	234/318 (0.74)	104/318 (0.33)	0/318 (0.00)
VUHD85	685/162 (4.23)	613/162 (3.78)	549/162 (3.39)	473/162 (2.92)	404/162 (2.49)	330/162 (2.04)	275/162 (1.70)	211/162 (1.30)	148/162 (0.91)	74/162 (0.46)	0/162 (0.00)
VUHD87	1/0 (-)	1/0 (-)	1/0 (-)	1/0 (-)	1/0 (-)	1/0 (-)	1/0 (-)	0/0 (-)	0/0 (-)	0/0 (-)	0/0 (-)
VUHD92	1791/434 (4.13)	1613/434 (3.72)	1416/434 (3.26)	1210/434 (2.79)	1050/434 (2.42)	888/434 (2.05)	703/434 (1.62)	533/434 (1.23)	346/434 (0.80)	178/434 (0.41)	0/434 (0.00)
VUHD94	3852/928 (4.15)	3463/928 (3.73)	3115/928 (3.36)	2709/928 (2.92)	2321/928 (2.50)	1943/928 (2.09)	1548/928 (1.67)	1143/928 (1.23)	773/928 (0.83)	418/928 (0.45)	0/928 (0.00)
VUHD98	2256/538 (4.19)	2048/538 (3.81)	1804/538 (3.35)	1600/538 (2.97)	1351/538 (2.51)	1137/538 (2.11)	932/538 (1.73)	713/538 (1.33)	457/538 (0.85)	220/538 (0.41)	0/538 (0.00)
donor 1	3873/956 (4.05)	3445/956 (3.60)	3073/956 (3.21)	2712/956 (2.84)	2325/956 (2.43)	1926/956 (2.01)	1553/956 (1.62)	1118/956 (1.17)	771/956 (0.81)	409/956 (0.43)	0/956 (0.00)
donor 2	15188/3744 (4.06)	13659/3744 (3.65)	12203/3744 (3.26)	10683/3744 (2.85)	9136/3744 (2.44)	7625/3744 (2.04)	6103/3744 (1.63)	4545/3744 (1.21)	3101/3744 (0.83)	1547/3744 (0.41)	0/3744 (0.00)
lafy_donor_1	349/86 (4.06)	328/86 (3.81)	289/86 (3.36)	244/86 (2.84)	205/86 (2.38)	175/86 (2.03)	139/86 (1.62)	106/86 (1.23)	69/86 (0.80)	33/86 (0.38)	0/86 (0.00)
lafy_donor_2	1145/328 (3.49)	1018/328 (3.10)	905/328 (2.76)	795/328 (2.42)	692/328 (2.11)	559/328 (1.70)	468/328 (1.43)	344/328 (1.05)	226/328 (0.69)	113/328 (0.34)	0/328 (0.00)
lafy_donor_3	1721/446 (3.86)	1557/446 (3.49)	1383/446 (3.10)	1209/446 (2.71)	1047/446 (2.35)	870/446 (1.95)	675/446 (1.51)	494/446 (1.11)	318/446 (0.71)	153/446 (0.34)	0/446 (0.00)
lafy_donor_5	1739/444 (3.92)	1554/444 (3.50)	1397/444 (3.15)	1223/444 (2.75)	1042/444 (2.35)	865/444 (1.95)	693/444 (1.56)	512/444 (1.15)	357/444 (0.80)	181/444 (0.41)	0/444 (0.00)

Supplementary Table 3: Classification patterns for HLCA core cell types, in the naive split setting. The slope values are the slope of the performance curve across proportions of female cells, on the male and female test set. Categorization is performed according to the slope test and flip test (see Methods for details). The last column shows the counts of each cell type in the male and female test set (formatted as 'counts in male test set / counts in female test set'). All values are rounded to the third decimal. In total, 24 'Distinct' cell types, 20 'Non-distinct' and 17 'Inconclusive'.

Cell type	Slope (male)	Slope (female)	Slope test	Flip test	Performance difference	Classification trend	Test set counts (M/F)
AT0	-0.108	0.154	True	True	0.311	Distinct	65 / 189
AT1	-0.000	0.007	True	True	0.007	Inconclusive	630 / 624

AT2	-0.005	0.007	True	True	0.010	Inconclusive	4105 / 6201
AT2 proliferating	-0.017	0.099	True	True	0.138	**Distinct**	44 / 128
Adventitial fibroblasts	-0.101	-0.035	False	True	0.084	**Non-distinct**	1169 / 275
Alveolar Mph CCL3+	-0.410	0.315	True	True	0.596	**Distinct**	937 / 180
Alveolar Mph MT-positive	0.867	0.597	False	False	0.421	**Non-distinct**	13 / 221
Alveolar Mph proliferating	-0.056	0.172	True	True	0.115	**Distinct**	89 / 76
Alveolar fibroblasts	0.013	0.146	False	True	0.152	**Non-distinct**	386 / 446
Alveolar macrophages	-0.032	0.016	True	True	0.042	**Distinct**	7369 / 4487
B cells	-0.004	-0.004	False	False	0.010	Inconclusive	375 / 328
Basal resting	-0.035	-0.003	False	True	0.041	Inconclusive	3235 / 2841
CD4 T cells	0.032	0.095	False	True	0.076	**Non-distinct**	1202 / 2418
CD8 T cells	-0.077	-0.023	False	True	0.064	**Non-distinct**	2323 / 2260
Classical monocytes	-0.006	0.075	True	True	0.068	**Distinct**	1231 / 1655
Club (nasal)	-0.041	0.057	True	True	0.100	**Distinct**	1709 / 2598
Club (non-nasal)	-0.045	0.124	True	True	0.128	**Distinct**	693 / 931
DC1	-0.113	0.014	True	False	0.216	**Non-distinct**	21 / 33
DC2	0.042	0.149	False	True	0.096	**Non-distinct**	685 / 778
Deuterosomal	-0.023	0.021	True	True	0.058	**Distinct**	75 / 87
EC aerocyte capillary	-0.027	-0.015	False	True	0.020	Inconclusive	768 / 256
EC arterial	-0.085	0.015	True	True	0.084	**Distinct**	763 / 310
EC general capillary	-0.018	0.021	True	True	0.047	**Distinct**	1685 / 631
EC venous pulmonary	-0.082	0.016	True	True	0.063	**Distinct**	669 / 260
EC venous systemic	0.005	0.073	False	True	0.055	Inconclusive	472 / 589
Goblet (bronchial)	-0.499	0.249	True	True	0.615	**Distinct**	150 / 104
Goblet (nasal)	-0.017	0.028	True	True	0.057	**Distinct**	2113 / 3933
Goblet (subsegmental)	-0.037	0.052	True	True	0.111	**Distinct**	69 / 88
Hematopoietic stem cells	0.364	0.795	False	False	0.417	**Non-distinct**	3 / 8
Hillock-like	-0.119	0.210	True	True	0.241	**Distinct**	277 / 496
Interstitial Mph perivascular	-0.247	-0.149	False	True	0.097	**Non-distinct**	519 / 167
Ionocyte	-0.027	-0.009	False	False	0.035	Inconclusive	48 / 39
Lymphatic EC differentiating	-0.586	-0.805	False	False	0.480	**Non-distinct**	69 / 7
Lymphatic EC mature	-0.010	0.008	True	True	0.012	Inconclusive	389 / 204
Lymphatic EC proliferating	0.000	0.000	False	False	0.000	Inconclusive	3 / 2
Mast cells	0.005	0.005	False	False	0.006	Inconclusive	344 / 798
Mesothelium	-0.887	-0.818	False	False	0.276	**Non-distinct**	29 / 1
Migratory DCs	-0.024	-0.058	False	False	0.146	**Non-distinct**	23 / 28
Monocyte-derived Mph	-0.169	0.149	True	True	0.259	**Distinct**	2681 / 1542
Multiciliated (nasal)	-0.161	0.250	True	True	0.361	**Distinct**	375 / 401
Multiciliated (non-nasal)	-0.027	-0.004	False	True	0.029	Inconclusive	2982 / 2482
Myofibroblasts	-0.077	0.244	True	True	0.223	**Distinct**	46 / 73
NK cells	0.003	0.049	False	True	0.047	Inconclusive	904 / 2012
Neuroendocrine	0.000	0.000	False	False	0.000	Inconclusive	11 / 15
Non-classical monocytes	-0.151	0.020	True	True	0.182	**Distinct**	853 / 462
Peribronchial fibroblasts	-0.044	0.113	True	True	0.080	**Distinct**	140 / 100
Pericytes	-0.001	-0.007	False	False	0.009	Inconclusive	321 / 116
Plasma cells	-0.000	-0.001	False	True	0.003	Inconclusive	116 / 177
Plasmacytoid DCs	-0.009	-0.055	False	False	0.051	**Non-distinct**	50 / 33
SM activated stress response	-0.437	0.158	True	True	0.498	**Distinct**	53 / 30
SMG duct	-0.252	-0.037	False	True	0.183	**Non-distinct**	115 / 79
SMG mucous	0.000	0.032	False	False	0.023	Inconclusive	42 / 43
SMG serous (bronchial)	-0.085	-0.056	False	True	0.078	**Non-distinct**	147 / 58
SMG serous (nasal)	0.075	0.020	False	False	0.149	**Non-distinct**	47 / 223
Smooth muscle	0.013	0.022	False	True	0.013	Inconclusive	270 / 364
Smooth muscle FAM83D+	-0.355	0.048	True	False	0.561	**Non-distinct**	10 / 51
Subpleural fibroblasts	-0.113	0.242	True	True	0.320	**Distinct**	25 / 18
Suprabasal	-0.108	0.150	True	True	0.216	**Distinct**	2968 / 3690
T cells proliferating	0.347	0.303	False	False	0.164	**Non-distinct**	16 / 76
Tuft	-0.293	-0.085	False	False	0.254	**Non-distinct**	14 / 11
Pre-TB secretory	-0.337	-0.116	False	False	0.284	**Non-distinct**	449 / 192

Supplementary Table 4: Classification patterns for HLCA core cell types, in the donor-based data split setting. The slope values are the slope of the performance curve across proportions of female cells, on the male and female test set. Categorization is performed according to the slope test and flip test (see Methods for details). The last column shows the counts of each cell type in the male and female test set (formatted as 'counts in male test set / counts in female test set'). In total, 2 'Distinct' cell types, 46 'Non-distinct' and 13 'Inconclusive'; we can see the apparent sex bias effect has disappeared.

Cell type	Slope (male)	Slope (female)	Slope test	Flip test	Performance difference	Classification trend	Test set counts (M/F)
AT0	-0.155	0.131	True	True	0.181	**Distinct**	20 / 196
AT1	-0.004	-0.029	False	False	0.038	**Non-distinct**	189 / 616
AT2	-0.005	0.001	True	False	0.023	Inconclusive	3025 / 9924
AT2 proliferating	0.047	0.074	False	False	0.069	**Non-distinct**	37 / 252
Adventitial fibroblasts	-0.093	-0.134	False	False	0.078	**Non-distinct**	150 / 660

Alveolar Mph CCL3+	-0.372	-0.235	False	False	0.225	**Non-distinct**	64 / 697
Alveolar Mph MT-positive	0.985	1.009	False	False	0.167	**Non-distinct**	6 / 20
Alveolar Mph proliferating	0.000	-0.060	False	True	0.068	Inconclusive	5 / 56
Alveolar fibroblasts	0.233	0.095	False	False	0.409	**Non-distinct**	16 / 621
Alveolar macrophages	-0.030	-0.018	False	False	0.036	**Non-distinct**	1187 / 4652
B cells	0.004	-0.005	True	False	0.021	Inconclusive	375 / 250
Basal resting	-0.012	-0.035	False	False	0.019	**Non-distinct**	6686 / 141
CD4 T cells	0.048	0.083	False	True	0.031	**Non-distinct**	530 / 2085
CD8 T cells	-0.066	-0.041	False	False	0.038	**Non-distinct**	4181 / 4435
Classical monocytes	0.039	0.123	False	False	0.164	**Non-distinct**	358 / 1847
Club (nasal)	-0.003	0.026	True	False	0.353	Inconclusive	3226 / 7
Club (non-nasal)	0.096	0.265	False	True	0.206	**Non-distinct**	1767 / 183
DC1	-0.155	-0.047	False	False	0.544	**Non-distinct**	10 / 64
DC2	0.075	0.171	False	True	0.093	**Non-distinct**	574 / 561
Deuterosomal	0.016	0.011	False	False	0.070	**Non-distinct**	161 / 42
EC aerocyte capillary	0.000	-0.149	False	False	0.282	Inconclusive	4 / 78
EC arterial	-0.011	0.010	True	False	0.048	Inconclusive	243 / 404
EC general capillary	-0.070	-0.003	False	True	0.086	Inconclusive	73 / 552
EC venous pulmonary	-0.142	-0.169	False	False	0.084	**Non-distinct**	119 / 400
EC venous systemic	0.103	0.179	False	False	0.107	**Non-distinct**	1202 / 228
Goblet (bronchial)	-0.576	0.264	True	True	0.531	**Distinct**	6 / 43
Goblet (nasal)	0.011	0.072	False	True	0.081	**Non-distinct**	3676 / 69
Goblet (subsegmental)	-0.053	0.318	True	False	0.651	**Non-distinct**	152 / 4
Hematopoietic stem cells	-	-	-	-	-	Inconclusive	0 / 0
Hillock-like	0.133	0.181	False	True	0.118	**Non-distinct**	52 / 148
Interstitial Mph perivascular	-0.183	-0.355	False	True	0.145	**Non-distinct**	74 / 286
Ionocyte	-0.011	0.000	False	False	0.016	Inconclusive	64 / 6
Lymphatic EC differentiating	-0.455	-0.523	False	False	0.875	**Non-distinct**	1 / 8
Lymphatic EC mature	-0.031	-0.012	False	False	0.021	**Non-distinct**	143 / 354
Lymphatic EC proliferating	-	-	-	-	-	Inconclusive	0 / 0
Mast cells	0.007	0.004	False	False	0.008	**Non-distinct**	195 / 2260
Mesothelium	-	-	-	-	-	Inconclusive	0 / 0
Migratory DCs	0.045	-0.021	True	False	0.391	**Non-distinct**	8 / 64
Monocyte-derived Mph	-0.114	0.024	True	False	0.170	**Non-distinct**	704 / 3300
Multiciliated (nasal)	0.302	0.970	False	True	0.599	**Non-distinct**	1428 / 3
Multiciliated (non-nasal)	-0.043	-0.039	False	False	0.038	**Non-distinct**	3923 / 685
Myofibroblasts	0.000	0.361	False	False	0.422	Inconclusive	4 / 58
NK cells	0.012	-0.007	True	False	0.041	Inconclusive	153 / 3198
Neuroendocrine	0.000	0.000	False	False	0.062	Inconclusive	16 / 7
Non-classical monocytes	-0.113	-0.099	False	False	0.027	**Non-distinct**	262 / 623
Peribronchial fibroblasts	0.074	0.395	False	False	0.382	**Non-distinct**	265 / 29
Pericytes	0.000	-0.114	False	False	0.250	Inconclusive	2 / 12
Plasma cells	0.017	0.002	False	False	0.013	**Non-distinct**	154 / 201
Plasmacytoid DCs	0.000	-0.124	False	False	0.091	Inconclusive	16 / 11
SM activated stress response	-0.855	-0.732	False	True	0.432	**Non-distinct**	5 / 125
SMG duct	-0.223	0.045	True	False	0.343	**Non-distinct**	208 / 46
SMG mucous	-	-	-	-	-	Inconclusive	107 / 0
SMG serous (bronchial)	-	-	-	-	-	Inconclusive	236 / 0
SMG serous (nasal)	-	-	-	-	-	Inconclusive	217 / 0
Smooth muscle	0.001	0.027	False	False	0.052	**Non-distinct**	167 / 305
Smooth muscle FAM83D+	0.277	0.295	False	False	0.289	**Non-distinct**	22 / 25
Subpleural fibroblasts	-0.061	0.182	True	False	0.524	**Non-distinct**	3 / 7
Suprabasal	-0.050	0.036	True	False	0.176	**Non-distinct**	4812 / 1679
T cells proliferating	0.221	0.223	False	False	0.221	**Non-distinct**	7 / 11
Tuft	-0.622	-0.163	False	True	0.443	**Non-distinct**	25 / 4
Pre-TB secretory	-0.642	-0.352	False	True	0.303	**Non-distinct**	399 / 192

Supplementary Table 5: In the donor-based setting, classification pattern (along with slope values and maximum difference in performance between the male and female test set) for the AT0, bronchial goblet, monocyte-derived Mph and Suprabasal cell types after fixing the sample counts in the training set. For AT0 and bronchial goblet cells, we also include results from the matching analysis. The slope values are the slope of the performance curve across proportions of female cells, on the male and female test set. Categorization is performed according to the slope test and flip test (see Methods for details). Fixing cell counts removes the sex-distinct classification effect for all cell types except bronchial goblet cells. Matching on confounders removes the sex-distinct classification effect for AT0 cells, and renders the trend on bronchial goblet cells inconclusive.

**Cell type**	**Slope (male)**	**Slope (female)**	**Slope test**	**Flip test**	**Performance difference**	**Classification trend**
AT0 (after fixing)	-0.038	-0.011	False	False	0.197	**Non-distinct**
AT0 (after matching, no fixing)	0.079	0.124	False	False	0.308	**Non-distinct**
Goblet (bronchial) (after fixing)	-0.704	0.255	True	True	0.5	**Distinct**
Goblet (bronchial) (after matching, no fixing)	0	0.716	-	False	0.625	Inconclusive
Monocyte-derived Mph (after fixing)	0.086	0.037	False	False	0.152	**Non-distinct**

Suprabasal	-0.038	-0.011	False	False	0.197	**Non-distinct**

Supplementary Table 6: In the naive split setting, classification pattern (along with slope values and maximum difference in performance between the male and female test set) for the CD4 T-cell sub-population, before and after fixing the sample counts in the training set. The slope values are the slope of the performance curve across proportions of female cells, on the male and female test set. Categorization is performed according to the slope test and flip test (see Methods for details). We see that removing abundance effects changes the categorization of the cell type.

Cell type	Slope (male)	Slope (female)	Slope test	Flip test	Performance difference	Classification trend
CD4 T cells (before fixing)	0.032	0.095	False	True	0.076	**Non-distinct**
CD4 T cells (after fixing)	-0.011	0.046	True	True	0.061	**Distinct**

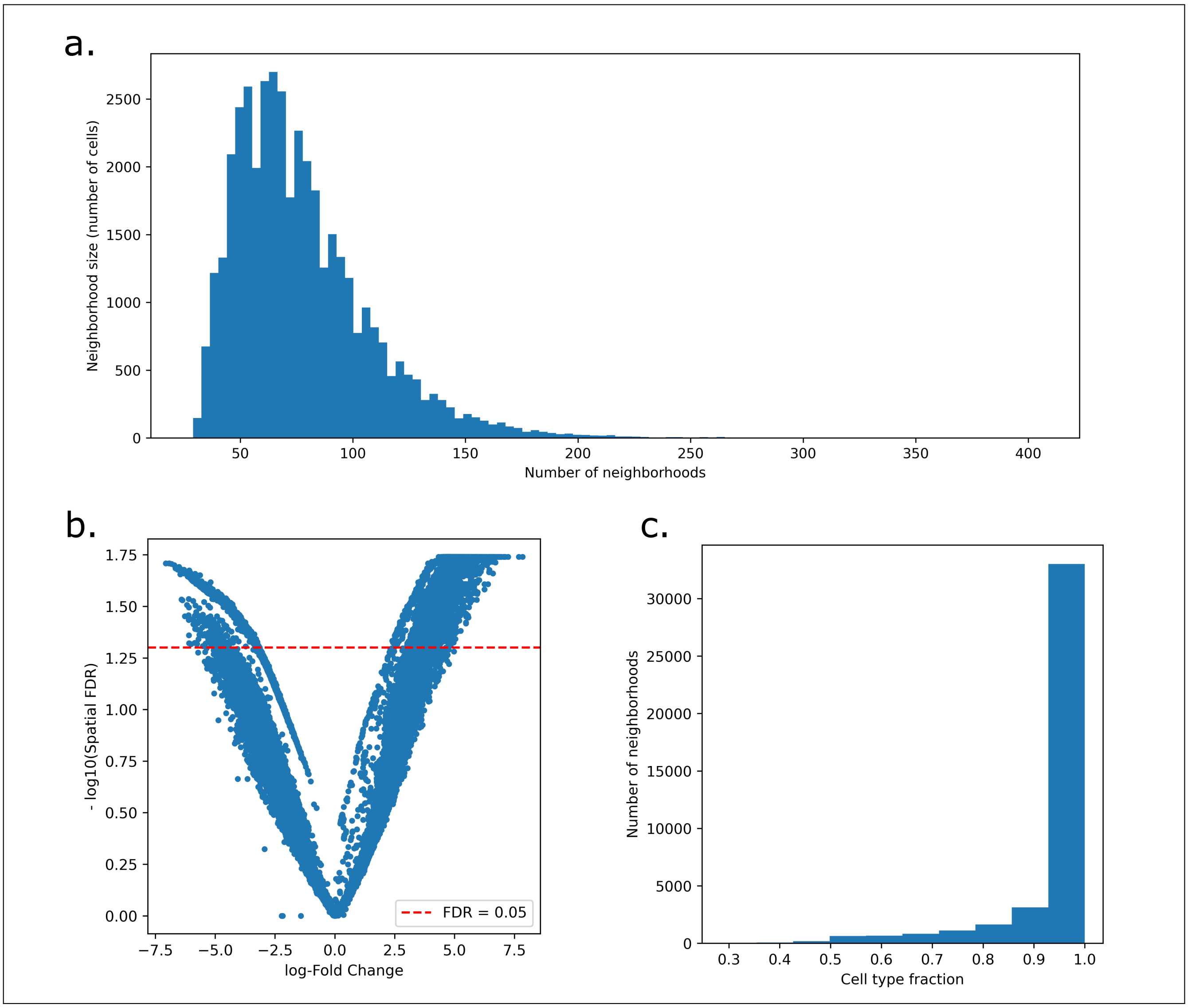

Supplementary Figure 1: Diagnostic plots for the differential abundance analysis. **a.** Distribution of neighborhood sizes. **b.** Significance (FDR value) vs Fold-Change, for each neighborhood. The red line indicates a significance threshold of 0.05. **c.** Distribution of neighborhoods in terms of the fraction of cells that correspond to the majority cell type label. For most cell types, almost all cells have the same annotation, indicating that neighborhoods have largely been assembled within existing cell type clusters.

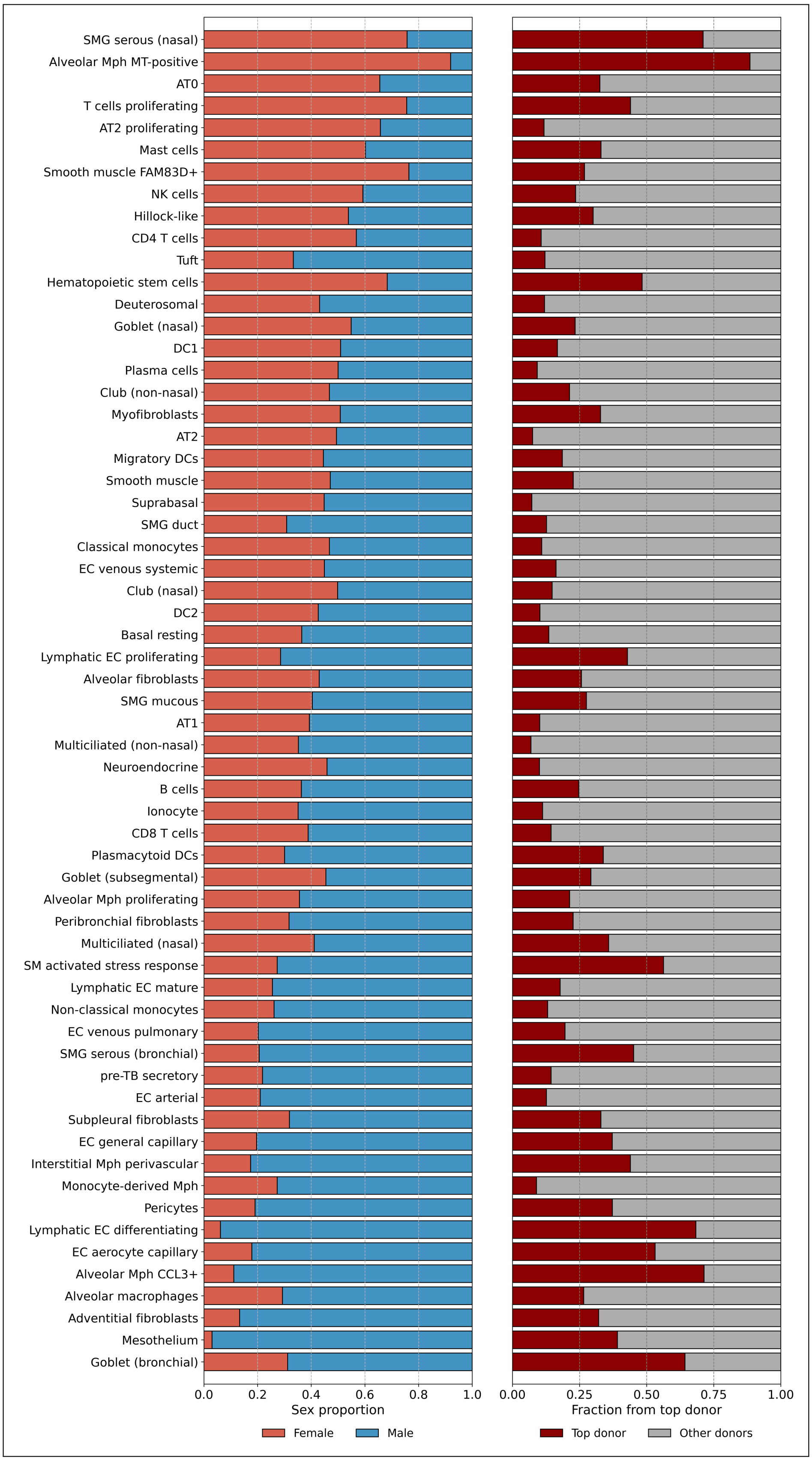

Supplementary Figure 2: Distribution of sex (left) and donors (right) among cell types in the HLCA core. Cell types are ordered to match Figure 1c. For 28 out of 61 cell types, one donor contributes over 25% of cells.

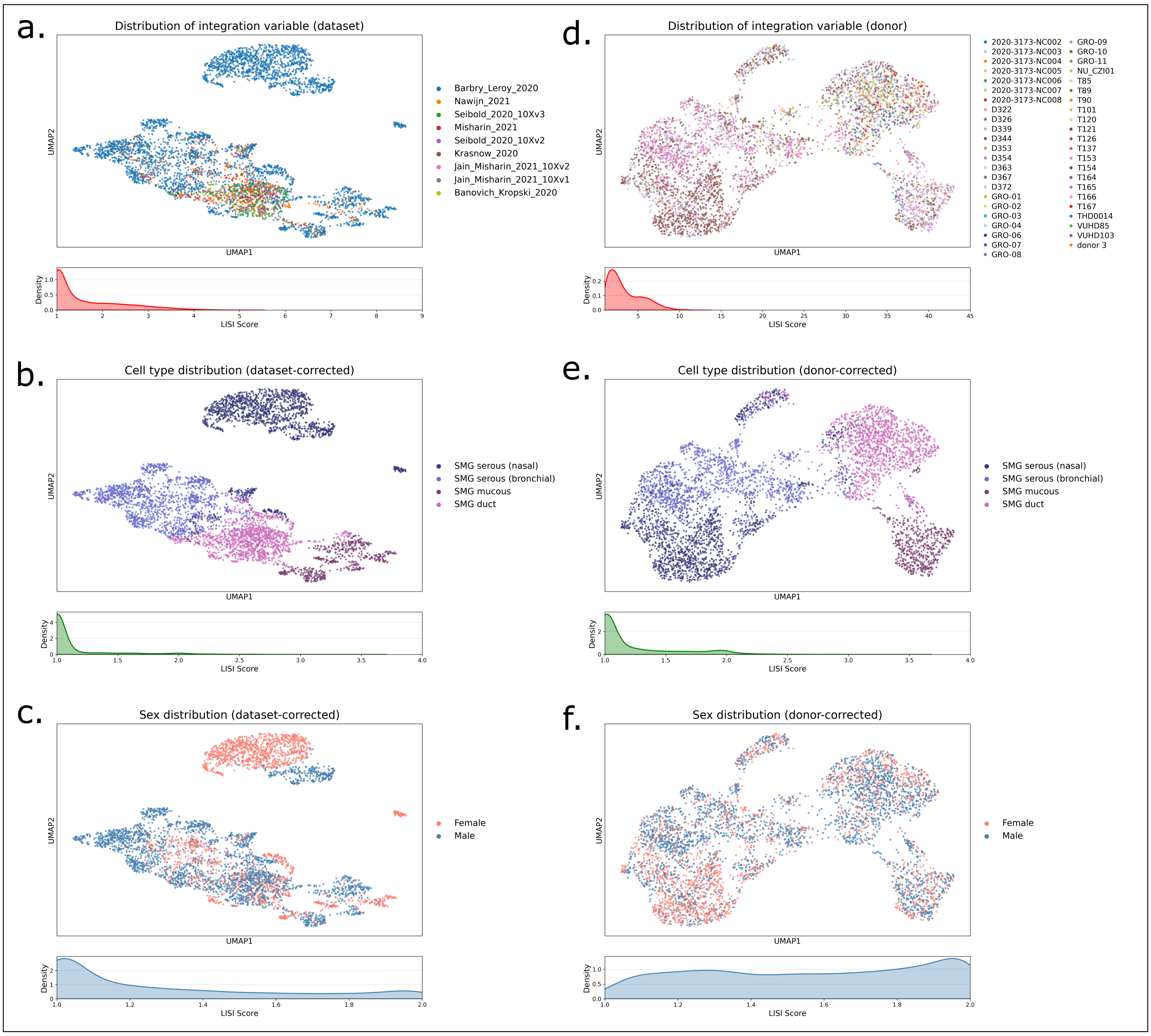

Supplementary Figure 3: Batch correction of the SMG population using Harmony, correcting for dataset of origin (**a-c**) and donor ID (**d-f**). UMAPs are colored by batch variable (a, d; donor IDs shortened in d for readability), cell type (b, e) and sex (c, f), with corresponding LISI score density plots shown below each UMAP. The LISI score ranges from 1 to the number of categories of the variable being assessed, where a score of 1 indicates clustering by the variable and the maximum value reflects complete mixing. Panels a, b, d, e are shown to verify the batch correction, panels c and f are of primary interest to evaluate sex-based clustering. In panels a and d, LISI scores are close to 1, which might suggest unsuccessful integration; however, LISI is known to underperform when category sizes are highly unbalanced, as is the case for both dataset and donor distributions in the SMG population. Visual inspection of the UMAP indicates no clear clustering by batch variable is present. Panels b and e confirm that cells continue to cluster by cell type after both corrections, indicating that biological information is sufficiently retained. In this case, the LISI score is reliable because subtype counts are well-balanced within the SMG data. Panels c and f reveal that sex-based clustering among SMG cells disappears with more stringent batch effect correction. After dataset-level correction, the LISI score is skewed towards 1, and the UMAP shows clear sex-distinct clusters in the nasal serous SMG cell type. By contrast, after donor-level correction, the LISI score distribution becomes more uniform, and distinct sex-based clusters are no longer apparent. This indicates that sex-based clustering in the SMG data may be a technical artifact of insufficient correction in the original HLCA integration.

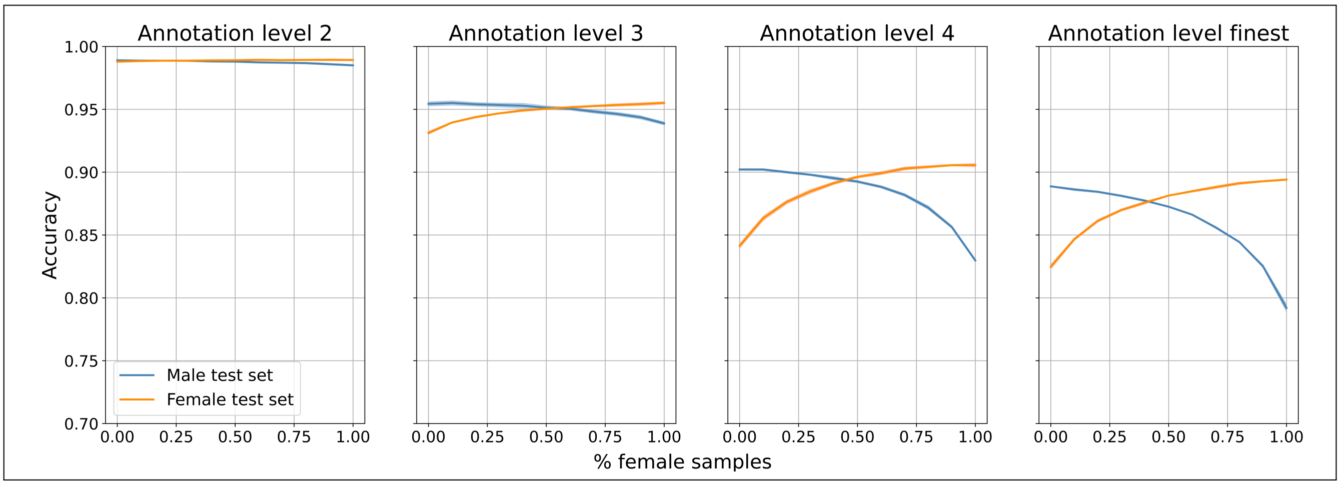

Supplementary Figure 4: Naive split setting, RF classifier - average accuracy on the male (blue) and female (orange) test set. X-axis shows the proportion of female cells in the training set, y-axis shows the accuracy score. The full line represents the mean accuracy across random seeds, the shaded area is the variance. Note that the trend is the same as for the KNN classifier (Figure 3c).

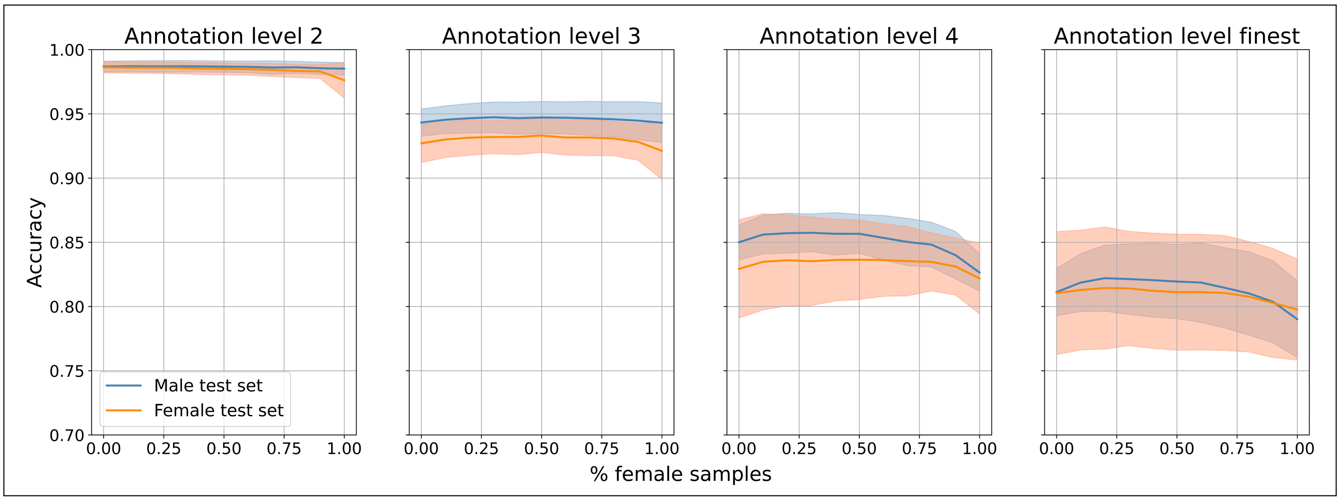

Supplementary Figure 5: Donor-based split setting, RF classifier - average accuracy on the male (blue) and female (orange) test set. X-axis shows the proportion of female cells in the training set, y-axis shows the accuracy score. The full line represents the mean accuracy across random seeds, the shaded area is the variance. Note the absence of a trend, as for the KNN classifier (Figure 3d).

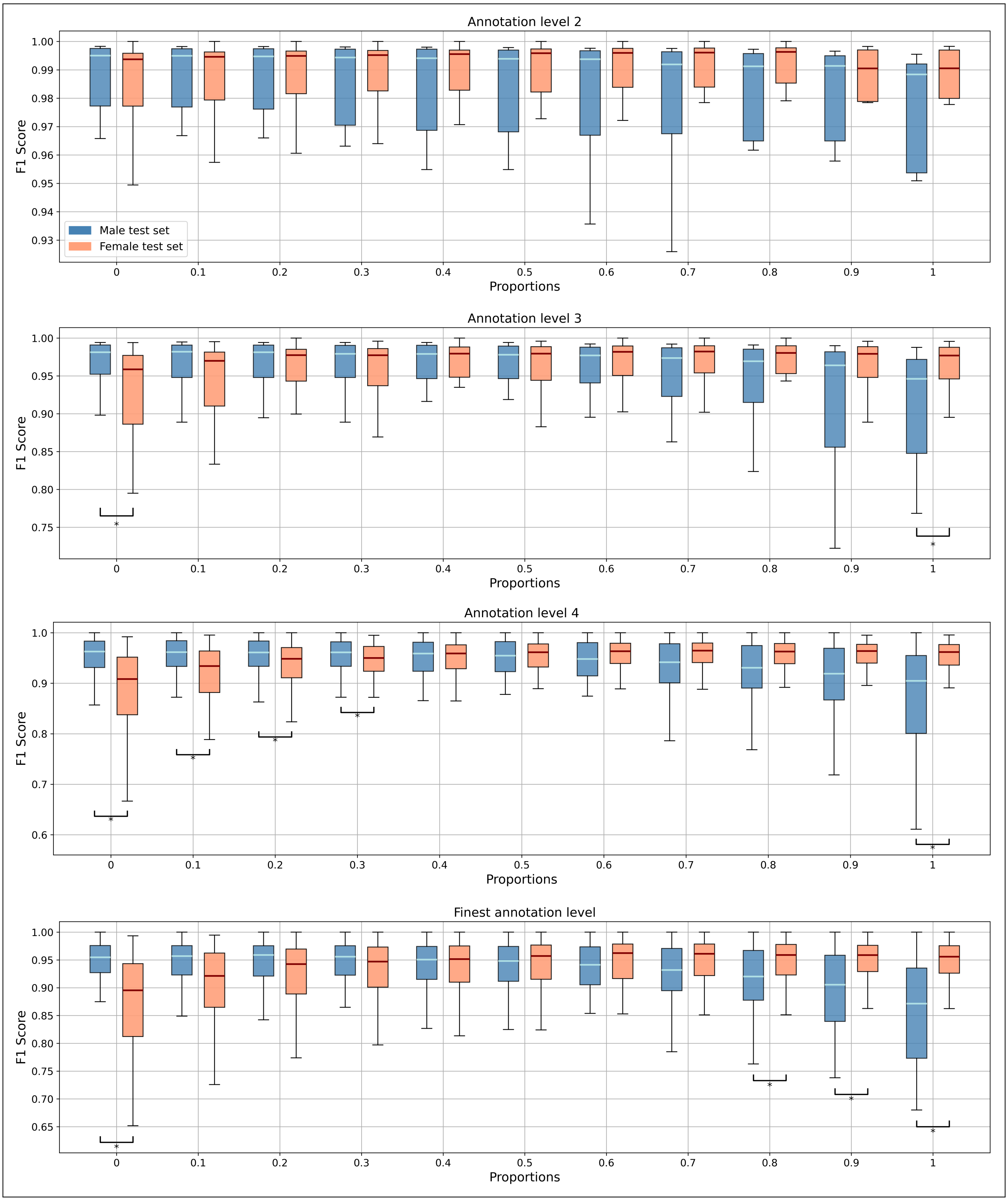

Supplementary Figure 6: Naive split setting, KNN classifier - distribution of F1 scores across classes, for annotation levels 2-5. Blue boxes denote assessment on the male test set, orange boxes on the female test set. Outliers are not plotted. Each point in a box corresponds to the F1 score on a cell type (the median across random seeds). The medians of each box are indicated by the horizontal lines (light blue for male and brown for female). Statistical significance, as calculated using a paired t-test, is denoted by '*' ($p < 5 \times 10^{-2}$); if not significant, no annotation is drawn. We can see that a significant gap in accuracy between the male and female test sets is only exhibited at extreme sex ratios (> 80% male or female).

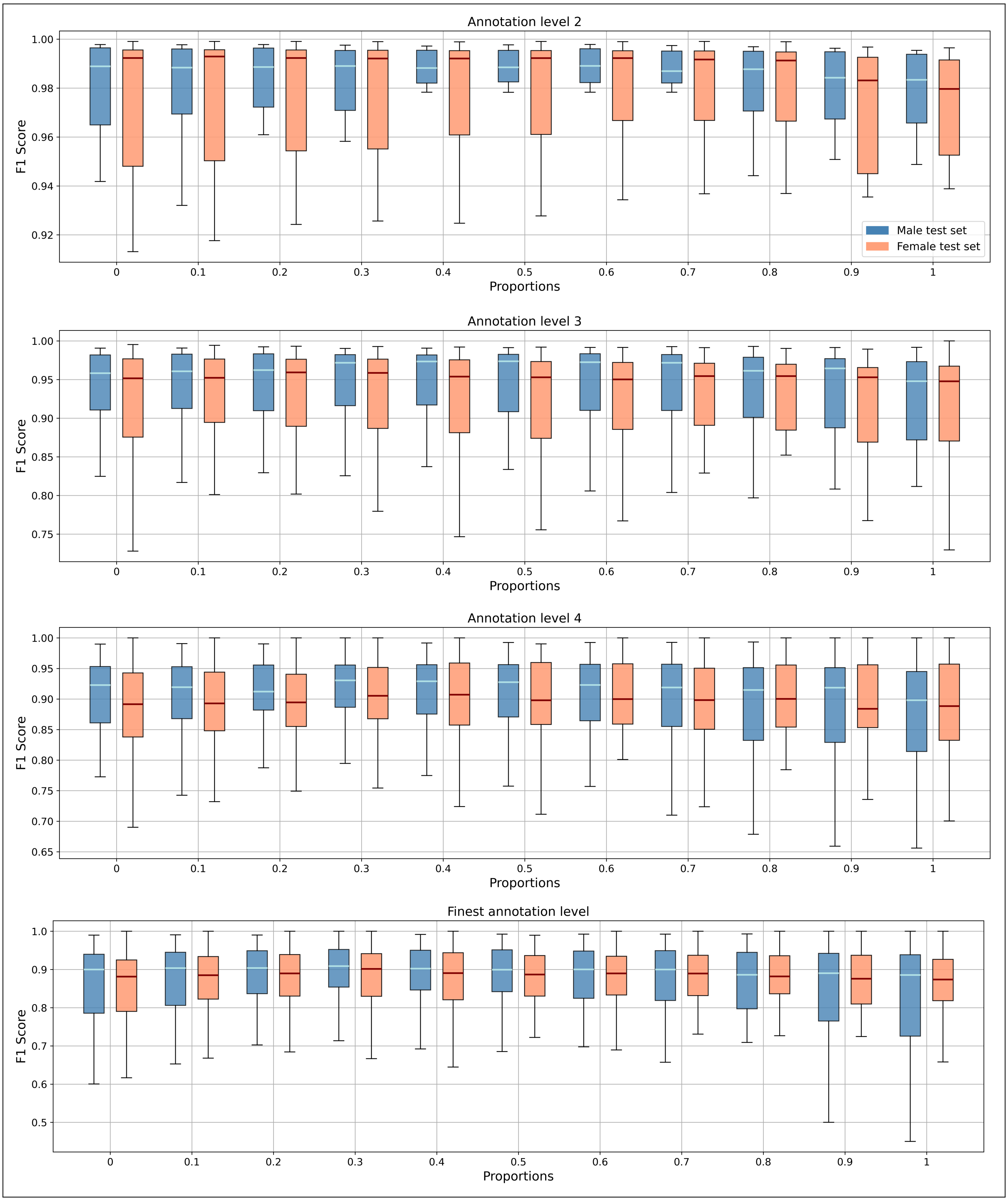

Supplementary Figure 7: Donor-based split setting, KNN classifier - distribution of F1 scores across classes, for annotation levels 2-5. Blue boxes denote assessment on the male test set, orange boxes on the female test set. Outliers are not plotted. Each point in a box corresponds to the F1 score on a cell type (the median across random seeds). The medians of each box are indicated by the horizontal lines (light blue for male and brown for female). Statistical significance could not be calculated as for the naive split setting, due to some classes missing for the male or the female test; however, we can see that there is no significant gap in accuracy between the male and female test sets, at any sex ratio.

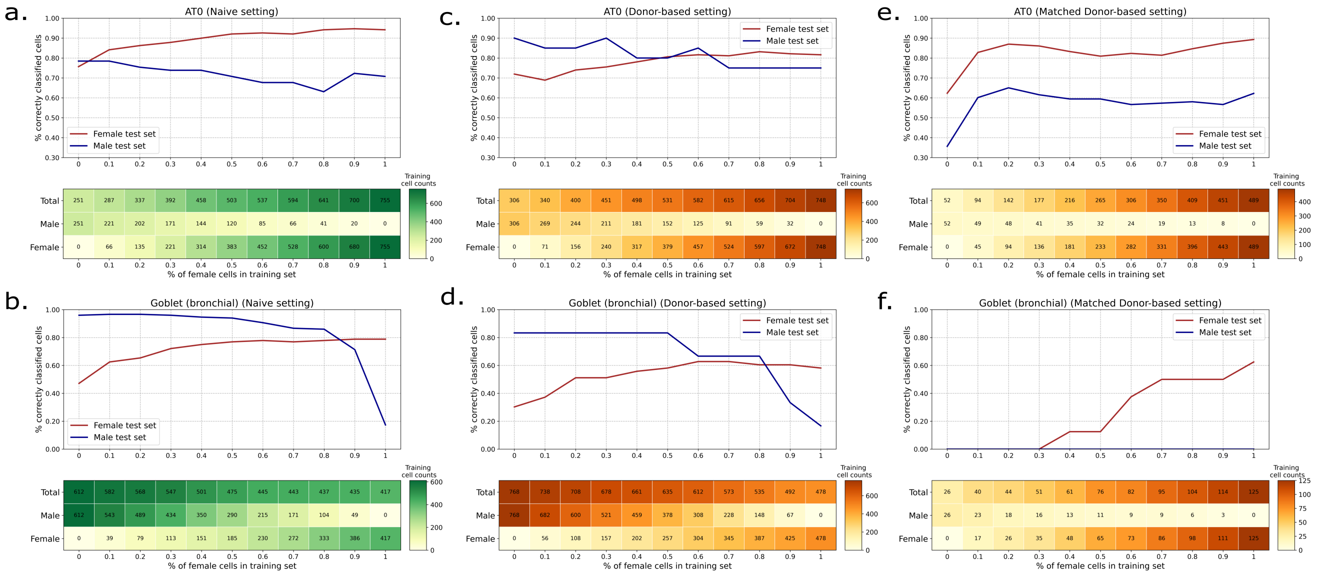

Supplementary Figure 8: Classifier behavior on individual cell types, where sex bias in the training set is amplified in both the naive and the donor-based setting, but is largely resolved under confounder matching. For every cell type, the line plot gives the proportion of correctly classified cells of that cell type in the female test set (red) and the male test set (blue), as the overall proportion of female cells in the training set increases. The heat map below each line plot gives the counts of that cell type in the training set (from top to bottom: total sample count, count of male samples and count of female samples). Results are shown for AT0 cells (**a, c, e**) and bronchial goblet cells (**b, d, f**) across three experimental designs: naive (**a-b**), donor-based (**c-d**) and matched donor-based (**e-f**), where individuals are matched on smoking status and tissue processing site. The classifier exhibits sex-distinct behavior in both the naive and the donor-based setting, for both AT0 cells (**a, c**) and bronchial goblet cells (**b, d**), indicating that donor-level stratification alone does not resolve the sex-based effect. However, in the matched donor-based setting, the sex-distinct trend disappears for AT0 cells (**e**), suggesting that confounders were causing a spurious association between sex and classifier performance on this cell type. For bronchial goblet cells (**f**), cell counts were too low after matching to draw reliable conclusions (classifier performance on the male test set is null regardless of the proportion of female cells in the training set).

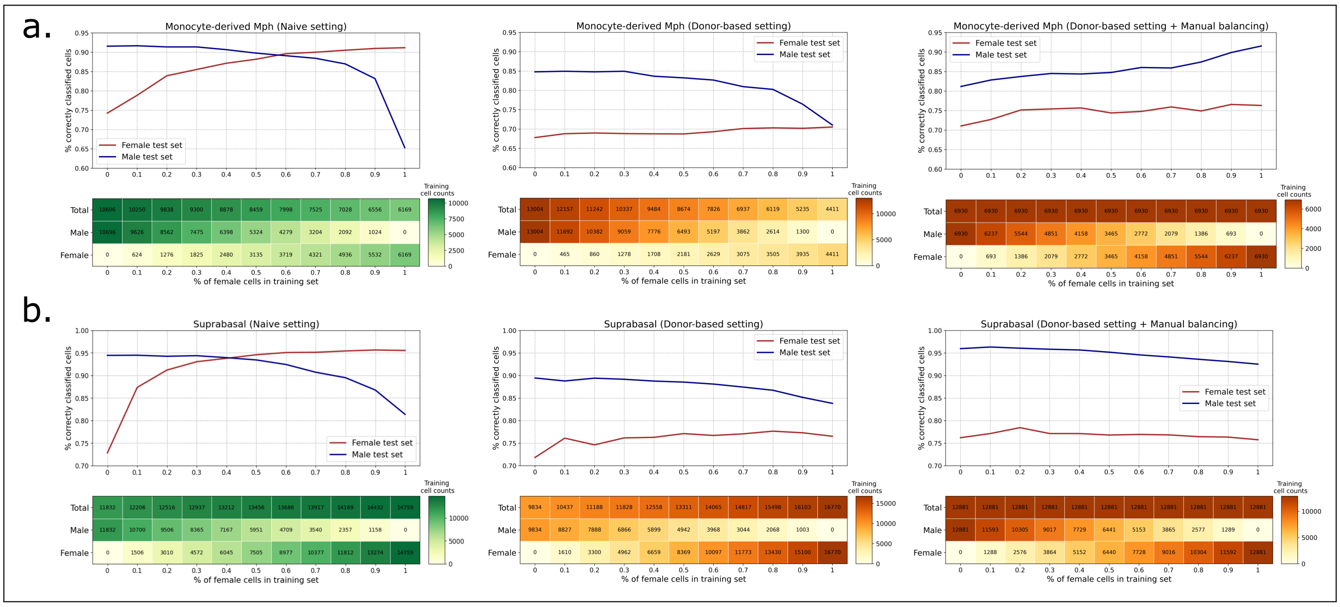

Supplementary Figure 9: Examples of classifier behavior on individual cell types, where sex bias in the training set is only amplified in the naive split setting, not in the donor-based setting. For every cell type, the line plot gives the proportion of correctly classified cells of that cell type in the female test set (red) and the male test set (blue), as the overall proportion of female cells in the training set increases. The heat map below each line plot gives the counts of that cell type in the training set (from top to bottom: total sample count, count of male samples and count of female samples). The third column shows results when the training counts of the cell type of interest are fixed. **a.** Monocyte-derived macrophage cells show sex-distinct classification behavior in the naive split setting (left), but disappears under donor-based splitting (middle) and even more so when we balance its counts in the training cells (right). **b.** Suprabasal cells, the sex-biased trend disappears as in a.

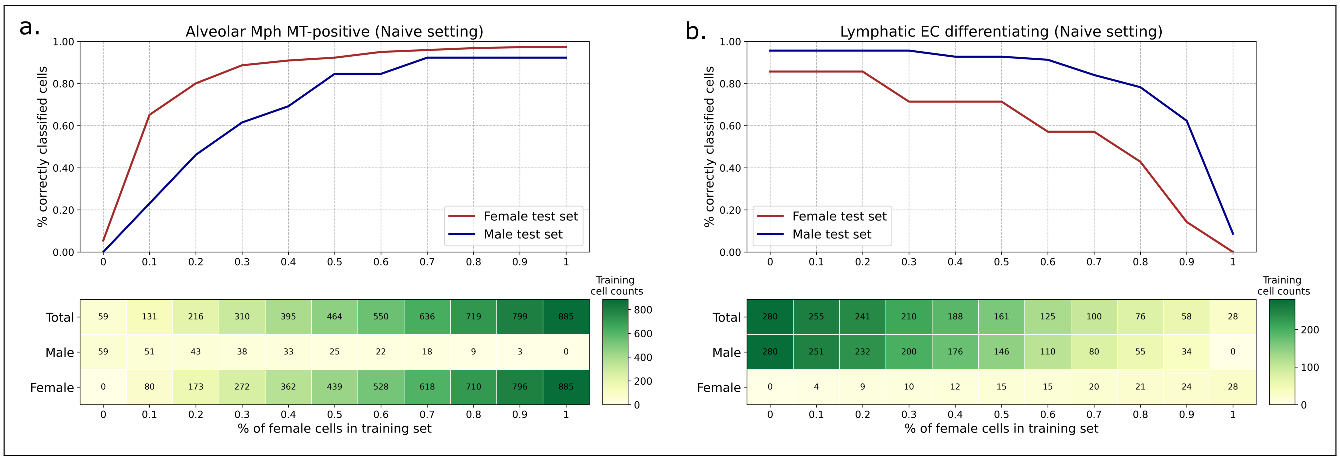

Supplementary Figure 10: In the naive split setting, cell types that exhibit the same classification trend on the male and female test set, likely due to abundance effects. For both cell types, the line plot gives the proportion of correctly classified cells of that cell type in the female test set (red) and the male test set (blue), as the overall proportion of female cells in the training set increases. The heat map below each line plot gives the counts of that cell type in the training set (from top to bottom: total sample count, count of male samples and count of female samples). **a.** Classifier behavior for MT-positive alveolar macrophages. The performance improves on both test sets as the proportion of female cells in the training set increases. Since MT-positive alveolar macrophages are female-dominated in terms of abundance (Figure 1c), as we increase the proportion of female cells, this cell type is sampled more, thus its count in the training set increases; this yields a better classifier performance, regardless of the test set. **b.** Classifier behavior for lymphatic endothelial cells. Reverse behavior compared to **a**: the performance decreases for both the male and the female test set, likely as a result of decreased counts in the training set because the cell type is male-dominated in terms of abundance (Figure 1c).

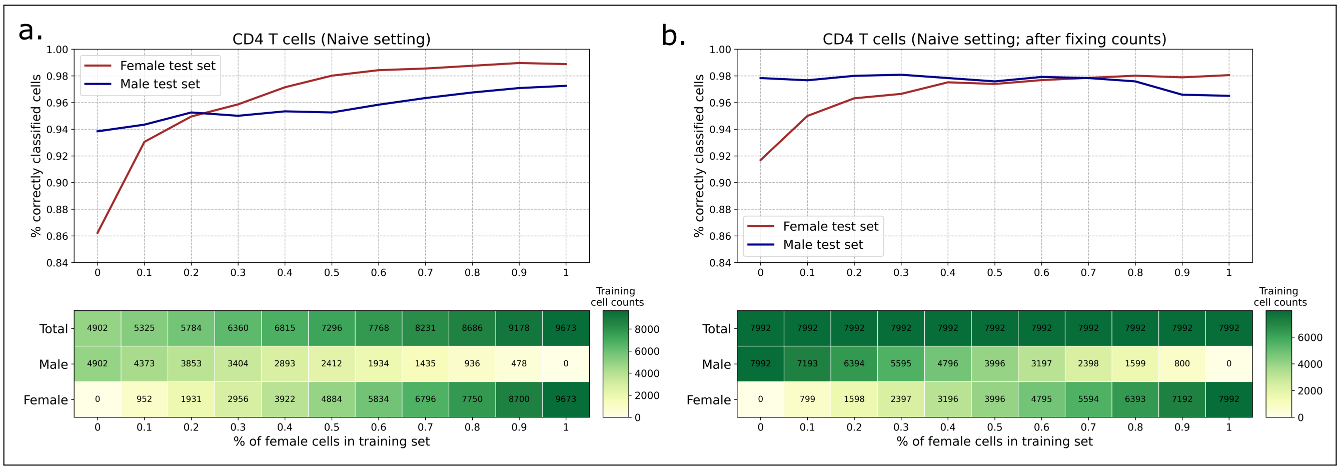

Supplementary Figure 11: In the naive split setting, classifier behavior on CD4 T-cells before (**a.**) and after (**b.**) fixing the sample counts in the training set. The line plots gives the proportion of correctly classified cells of that cell type in the female test set (red) and the male test set (blue), as the overall proportion of female cells in the training set increases. The heat map below each line plot gives the counts of that cell type in the training set (from top to bottom: total sample count, count of male samples and count of female samples). The classification trend is non-distinct in **a.** (due to the increasing male curve) and distinct in **b.**. This indicates that abundance differences indeed obscure distinct behavior of cell types in the naive split setting.

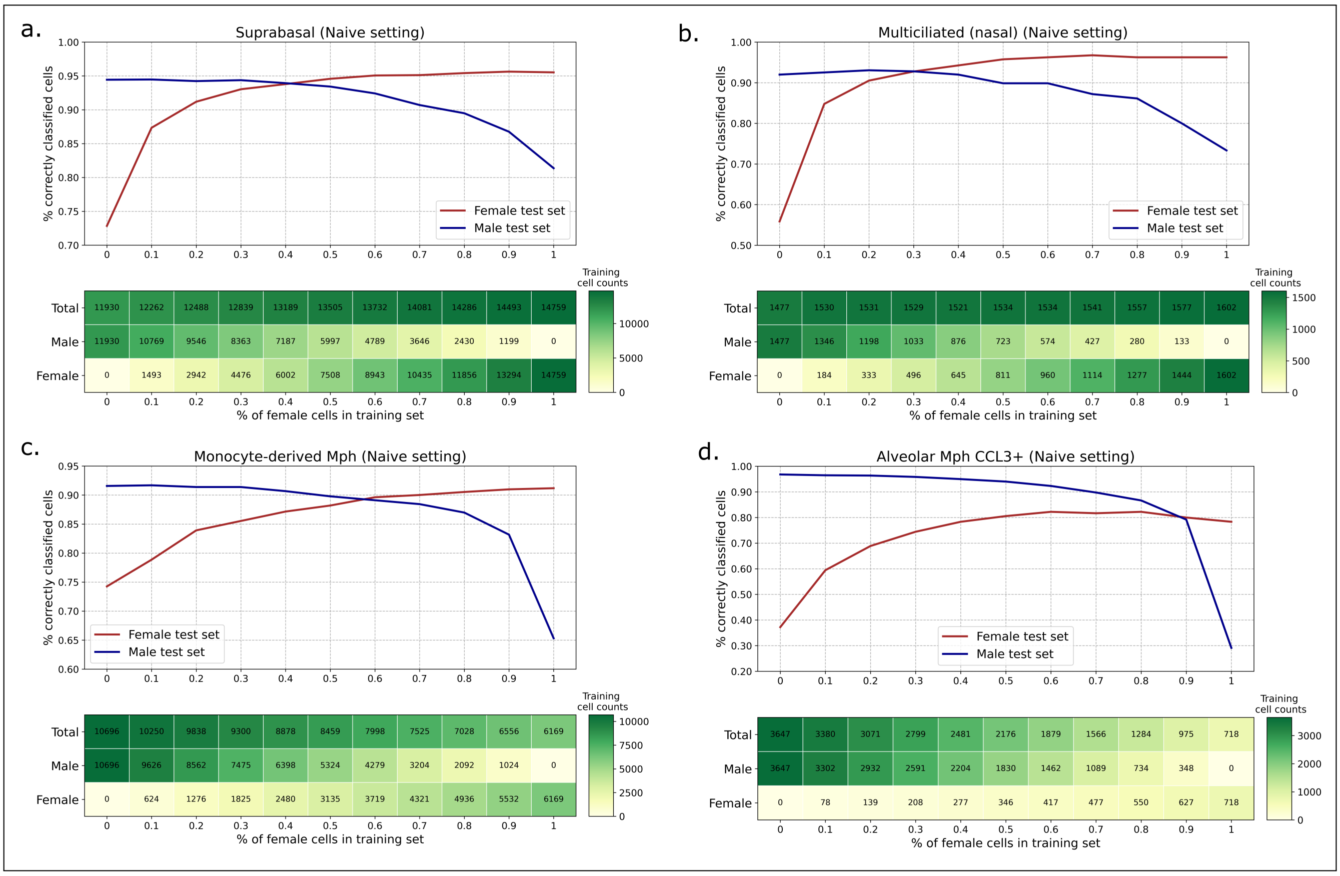

Supplementary Figure 12: In the naive split setting, cell types that exhibit a distinct (apparently sex-biased) classification trend on the male and female test set: suprabasal cells (**a.**), nasal multiciliated cells (**b.**), monocyte-derived macrophages (**c.**) and alveolar CCL3+ macrophages (**d.**). For all cell types, the line plot gives the proportion of correctly classified cells of that cell type in the female test set (red) and the male test set (blue), as the overall proportion of female cells in the training set increases. The heat map below each line plot gives the counts of that cell type in the training set (from top to bottom: total sample count, count of male samples and count of female samples). We see that the distinct effect can occur both for cell types that are balanced in terms of counts (**a.**, **b.**; total training set count does not vary significantly as we vary the proportion of female cells, top row of the heat map) and cells with large abundance differences (**c.**, **d.**).

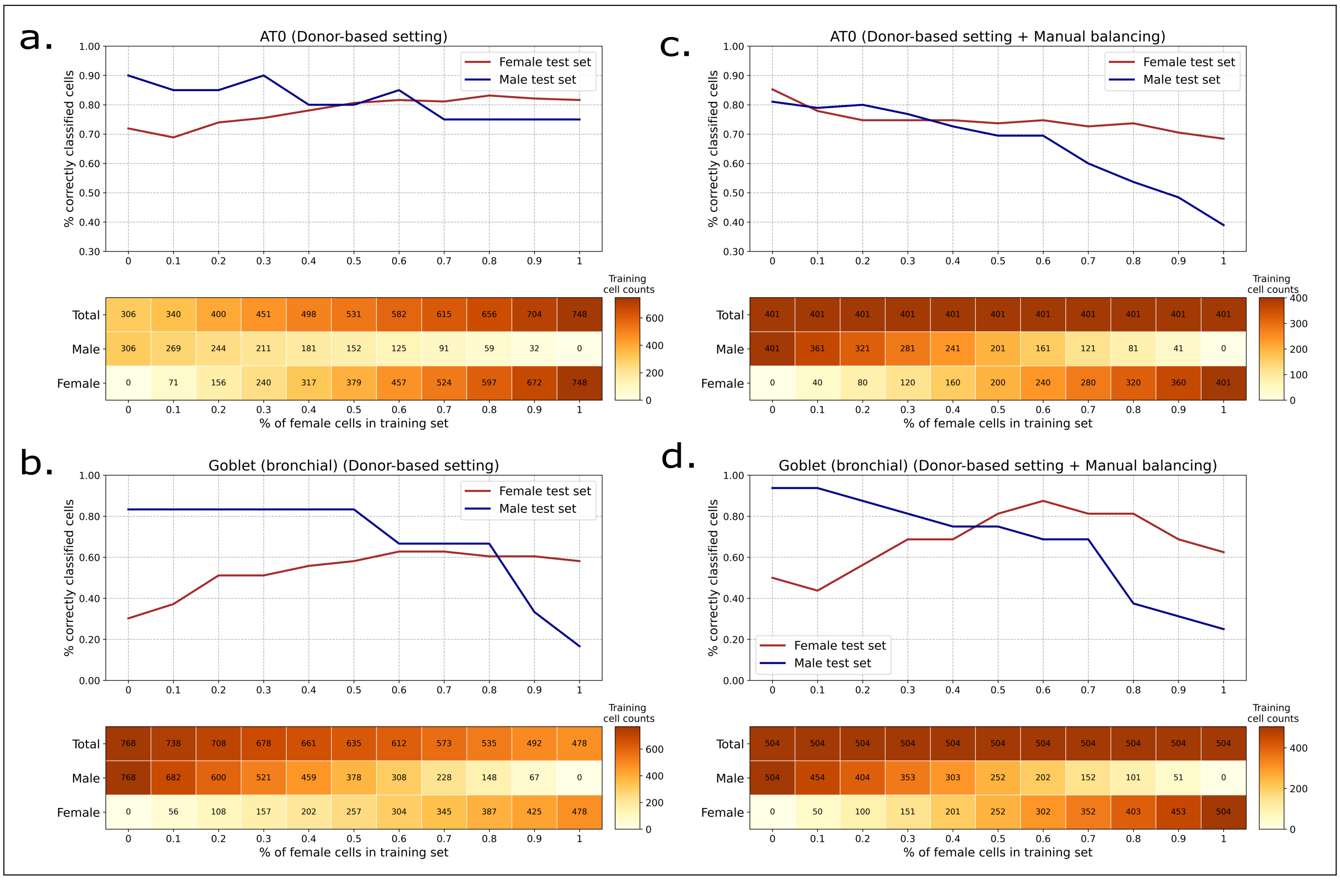

Supplementary Figure 13: In the donor-based split setting, classifier behavior before (**a,b**) and after (**a,c**) fixing the counts in the training set on AT0 cells and goblet bronchial cells. For both cell types, the line plot gives the proportion of correctly classified cells of that cell type in the female test set (red) and the male test set (blue), as the overall proportion of female cells in the training set increases. The heat map below each line plot gives the counts of that cell type in the training set (from top to bottom: total sample count, count of male samples and count of female samples). Before fixing cell counts, the classification trend is distinct for both AT0 cells (**a**) and bronchial goblet cells (**b**). After fixing cell counts, AT0 cells show a non-distinct trend (**c**), due to the non-increasing female curve, while the trend on bronchial goblet cells is still distinct (**d**).
